# Uncovering the  Kagome Ferromagnet within a Family of Metal–Organic Frameworks

**DOI:** 10.1021/acs.chemmater.2c00289

**Published:** 2022-06-09

**Authors:** Samuel A. Ivko, Katherine Tustain, Tristan Dolling, Aly Abdeldaim, Otto H. J. Mustonen, Pascal Manuel, Chennan Wang, Hubertus Luetkens, Lucy Clark

**Affiliations:** † School of Chemistry, University of Birmingham, Birmingham B15 2TT, U.K.; ‡ Department of Chemistry and Materials Innovation Factory, University of Liverpool, Liverpool L7 3NY, U.K.; § ISIS Neutron and Muon Source, Rutherford Appleton Laboratory, Didcot OX11 0QX, U.K.; ∥ Swiss Muon Source, Paul Scherrer Institut, Villigen 5232, Switzerland

## Abstract

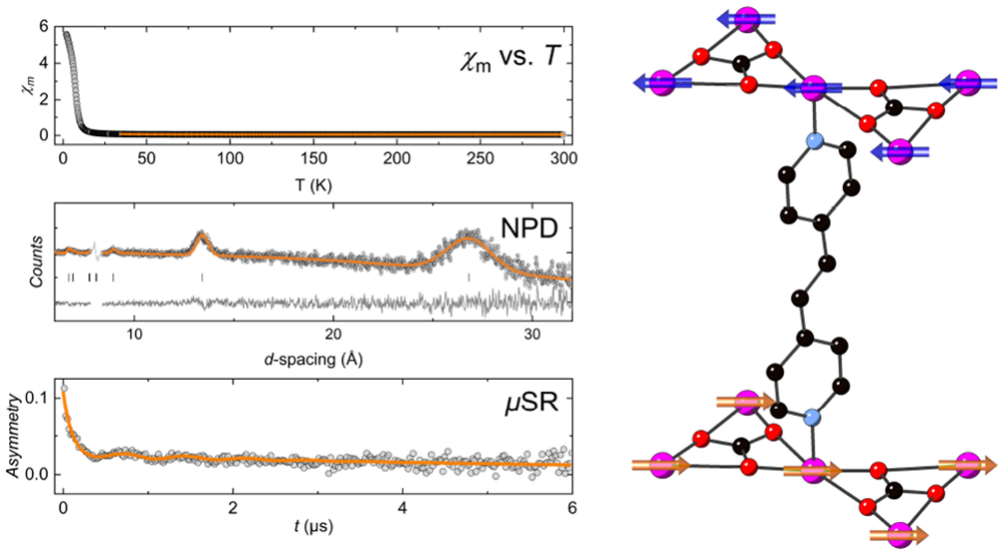

Kagome networks of ferromagnetically or antiferromagnetically coupled  magnetic moments represent important models in the pursuit of a diverse array of novel quantum and topological states of matter. Here, we explore a family of Cu^2+^-containing metal–organic frameworks (MOFs) bearing  kagome layers pillared by ditopic organic linkers with the general formula Cu_3_(CO_3_)_2_(*x*)_3_·2ClO_4_ (MOF-*x*), where *x* is 1,2-bis(4-pyridyl)ethane (*bpe*), 1,2-bis(4-pyridyl)ethylene (*bpy*), or 4,4′-azopyridine (*azpy*). Despite more than a decade of investigation, the nature of the magnetic exchange interactions in these materials remained unclear, meaning that whether the underlying magnetic model is that of an  kagome ferromagnet or antiferromagnet is unknown. Using single-crystal X-ray diffraction, we have developed a chemically intuitive crystal structure for this family of materials. Then, through a combination of magnetic susceptibility, powder neutron diffraction, and muon-spin spectroscopy measurements, we show that the magnetic ground state of this family consists of  ferromagnetic kagome layers that are coupled antiferromagnetically via their extended organic pillaring linkers.

## Introduction

It is widely appreciated that low-dimensional and frustrated exchange interactions in quantum magnetism give rise to exotic behaviors in solid-state materials.^1−6^ A model system in which these effects are manifested is the  kagome antiferromagnet, a frustrated two-dimensional network of corner-sharing equilateral triangles of antiferromagnetically coupled quantum  moments.^7^ From a theoretical perspective, the importance of this system is that it has been predicted to host a variety of novel quantum states of matter, including long-sought quantum spin liquid (QSL) states, due to the combination of the two-dimensional magnetic sublattice, a small spin magnitude, and geometric frustration.^8^ A widely studied material realization of the *S* =  kagome antiferromagnet is the inorganic material herbertsmithite, ZnCu_3_(OH)_6_Cl_2_, whose structure features a quasi-two-dimensional kagome network of antiferromagnetically coupled Cu^2+^ ions separated by diamagnetic Zn^2+^ ions^9^ that reveals many hallmarks of a QSL phase.^10,11^ More recently, there has also been growing interest in kagome ferromagnets, with investigations of layered materials such as Fe_3_Sn_2_^12^ and Co_3_Sn_2_S_2_^13^ demonstrating a diverse array of intriguing properties of materials. This includes the formation of topological magnon bands,^14^ the demonstration of spin–orbit torque,^15^ and the observation of giant anomalous Hall^13,16,17^ and Nernst effects.^18^ They have also been proposed as ideal systems in which to create and control the movement of skyrmions,^19,20^ which hold technological promise for future low-energy data storage.^21^

In the hunt for such novel phenomena of materials, more attention is being paid to magnetic inorganic–organic hybrid systems as promising alternatives to traditionally more widely studied inorganic compounds.^22−27^ For instance, in the search for unambiguous material realizations of  kagome magnets, the ability to separate inorganic kagome layers with organic components^28^ is an appealing material design strategy for overcoming the issue of magnetic site disorder that is common in purely inorganic systems.^29−31^ Metal–organic frameworks (MOFs) make up one such class of inorganic–organic hybrid materials, consisting of metal nodes joined by multitopic organic linkers to form (often porous) crystalline structures.^32^

The first reported example of a MOF containing  kagome layers within its crystal structure was the system known as Cu(1,3-*bdc*), where 1,3-*bdc* = 1,3-benzenedicarboxylate.^33^ In this material, each carboxylate group in the 1,3-*bdc* linker bridges two Cu^2+^ ions to form  kagome planes, which are layered into a three-dimensional crystal structure due to the ditopic nature of the 1,3-*bdc* linker, which also allows it to act as a pillar for the kagome layers. Cu(1,3-*bdc*) was found to exhibit strong antiferromagnetic interactions via Curie–Weiss analysis of magnetic susceptibility data, with a Weiss temperature (θ_CW_) of −33 K.^33^ However, the magnetic susceptibility data reported for this system also show evidence of a ferromagnetic transition in Cu(1,3-*bdc*) upon cooling. This is further evidenced by hysteretic behavior in the magnetization versus field data at 2 K with a coercive field of 1.05 mT and a singularity in the magnetic heat capacity at 2 K.^33^ Thus, it was initially hypothesized that the carboxylate-mediated in-plane superexchange interaction between nearest-neighbor Cu^2+^ moments in the kagome layers of Cu(1,3-*bdc*) is antiferromagnetic, and that the ferromagnetic component driving long-range magnetic order is derived from a seven-atom superexchange pathway through the pillaring 1,3-*bdc* linker between the kagome layers.^33^ This hypothesis was supported by models of other copper-containing systems with similar superexchange geometries.^34,35^ However, neutron scattering studies subsequently revealed the presence of ferromagnetic kagome layers in Cu(1,3-*bdc*), weakly coupled via an antiferromagnetic interaction along the seven-atom superexchange pathway between Cu^2+^ ions in adjacent layers.^14^ Importantly, this clearly demonstrates that magnetic exchange can be facilitated through extended organic moieties in MOF kagome systems and that understanding the exchange pathways and interactions in such materials may not be as straightforward to infer through the semiempirical Goodenough–Kanamori rules successfully developed and applied to purely inorganic systems.^36−38^

**Figure 1:**
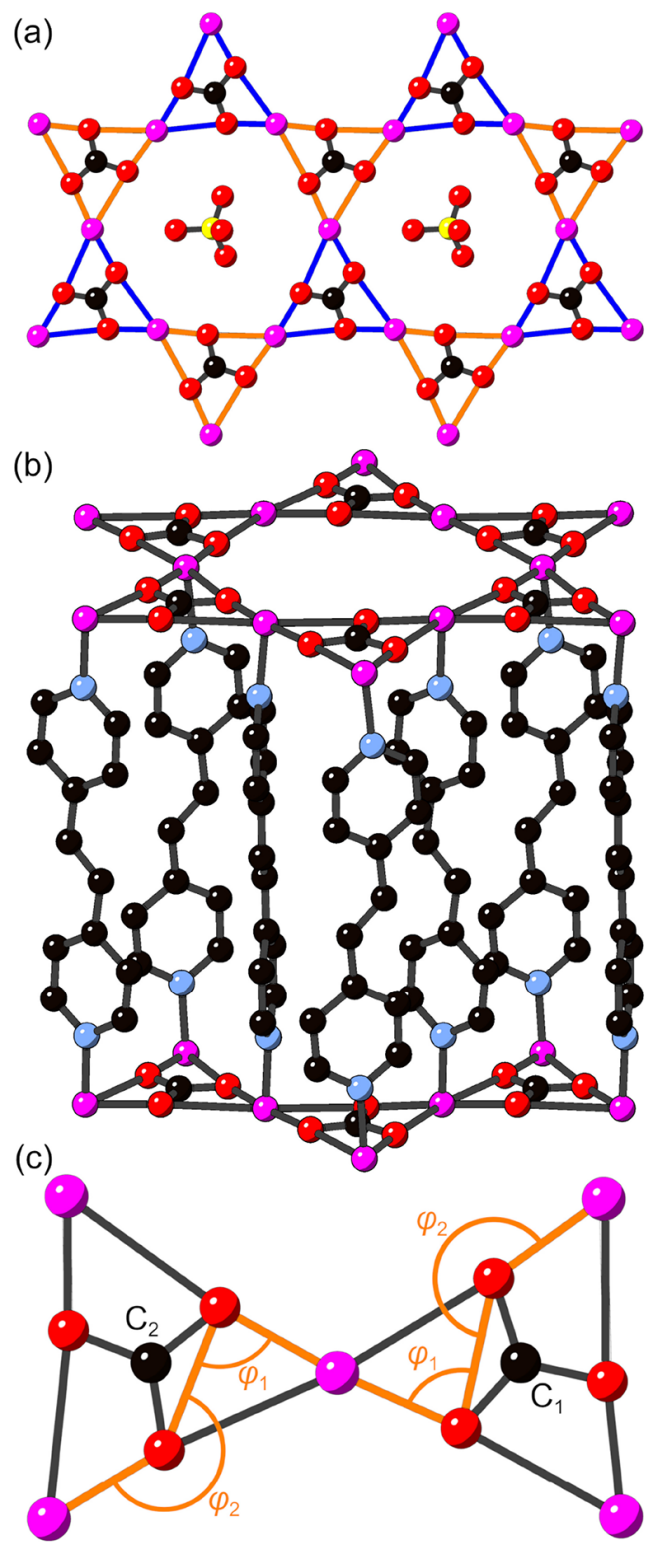
(a) Kagome layer in MOF-*bpe* showing one of the disordered perchlorate anions occupying the hexagonal channels, where blue and orange lines show the two differently sized triangles within the breathing kagome network (pillaring linkers omitted for the sake of clarity). (b) Mode of pillaring as seen in MOF-*bpy* (only one disordered linker orientation shown, hydrogen atoms omitted). (c) Demonstration of the Cu–O···O angles pertinent to superexchange between adjacent copper ions in the carbonate-chelated kagome network (perchlorate anions and linker atoms omitted for the sake of clarity; Cu, magenta; C, black; O, red; Cl, yellow; N, light blue).

However, one particularly valuable opportunity offered through the exploration of MOF realizations of  kagome magnets is the ability to tune or control the magnetic exchange interactions within or between the kagome layers by varying the organic linker molecules within the structure.^39−41^ A particularly interesting family of  kagome MOFs in this regard is that with the general formula Cu_3_(CO_3_)_2_(*x*)_3_·2ClO_4_ (MOF-*x*), which has been reported for a variety of pillaring linkers *x* = 1,2-bis(4-pyridyl)ethane (*bpe*),^42^ 1,2-bis(4-pyridyl)ethylene (*bpy*),^39^ or 1,2-bis(4-pyridyl)acetylene (*bpa*).^43^ In these materials, Cu^2+^ ions are coordinated to tridentate tris-chelated carbonate ligands in the crystallographic *a–b* plane (Figure 1a) to form kagome layers that are pillared along the *c* axis by a ditopic organic linker, *x* (Figure 1b). The hexagonal channels within the kagome layers are occupied by charge-balancing perchlorate anions that are subject to substantial disorder about the symmetry elements due to their weak interactions with the framework.

In the structure of MOF-*bpe*, reported in the hexagonal *P*6̅ space group,^42^ there is further disorder in the orientation of the *bpe* linkers in the framework along the *c* axis. As in Cu(1,3-*bdc*), initial Curie–Weiss analysis of magnetic susceptibility data for MOF-*bpe* indicated net antiferromagnetic interactions with a θ_CW_ of −2.13 K, but with a large upturn in the data below 12 K indicative of ferromagnetic interactions at low temperatures, too.^42^ From the approximately 180° Cu–O–Cu angles between the Cu^2+^ ions and carbonate oxygen atoms in the kagome layers, it was proposed that the in-plane exchange interactions in MOF-*bpe* are antiferromagnetic according to the Goodenough–Kanamori rules,^36−38^ and therefore, it was surmised that the ferromagnetic interactions indicated in the magnetic susceptibility data derive from coupling between layers, via a 10-atom superexchange pathway along the *bpe* pillars.^42^ However, subsequent density functional theory (DFT) calculations of tridentate μ_3_-CO_3_ Cu^2+^ units, comparable to those found in MOF-*bpe*, indicate that such an application of the Goodenough–Kanamori rules is insufficient to understand the exchange interactions between neighboring moments and that the strength and nature of the interactions are instead dependent on the Cu–O···O angles across the carbonate ligand, denoted as φ_1_ and φ_2_ (Figure 1c).^44^ In the reported crystal structure of MOF-*bpe*,^42^ the values of φ_1_ and φ_2_ are 82.6(3)° and 217.4(3)°, and 78.5(3)° and 221.5(3)°, respectively, for the two crystallographically independent carbonate ligands within the kagome layers. According to the DFT calculations,^44^ these Cu–O···O angles should result in a ferromagnetic exchange interaction on the order of 28 K between neighboring Cu^2+^ ions within the kagome layers.

More recent investigations of the magnetic properties of MOF-*bpe* have sparked further intrigue, as magnetic heat capacity data do not present a sharp anomaly associated with long-range magnetic order,^45^ despite magnetic susceptibility, ^1^H nuclear magnetic resonance (NMR), and high-field electron spin resonance (ESR) studies indicating ferromagnetic order below *T*_C_ ≈ 12 K.^39,42,45^ It has since been hypothesized that MOF-*bpe* exhibits short-range canted antiferromagnetic order, with competing ferromagnetic and antiferromagnetic interactions within the kagome layers, based on AC magnetic susceptibility measurements and the absence of magnetic Bragg peaks in neutron powder diffraction (NPD) data.^46^ As such, the true nature of the magnetic ground state of MOF-*bpe* is unclear.

In addition, there are also conflicting reports regarding the magnetic properties of several other members of the Cu_3_(CO_3_)_2_(*x*)_3_·2ClO_4_ family of  kagome magnets. For instance, in a study comparing MOF-*bpe* with MOF-*bpy*,^39^ the magnetic transition temperatures for each system were measured as 5.7 and 9.3 K, respectively. This was rationalized as being driven by the enhanced ability of *bpy* to facilitate the ferromagnetic exchange between the kagome layers compared with that of *bpe*, due to its increased rigidity and electronic delocalization. However, this assessment should be treated with caution, as a range of *T*_C_ values have been reported for MOF-*bpe* from 5.7 K^39^ to 7 K^45^ and 12 K.^42^ Moreover, the magnetic properties described in the literature for MOF-*bpa* contrast with reports for MOF-*bpe* and -*bpy*.^43^ Magnetic susceptibility data for MOF-*bpa* indicate dominant ferromagnetic interactions with a θ_CW_ of 26.6 K, and magnetization versus field data exhibit a linear increase in moment with increasing field up to 0.015 T, after which the gradient increases sharply until saturation is achieved at 0.5 T.^43^ It is argued that this field-dependent switching behavior is characteristic of a metamagnet with ferromagnetic layers aligning antiferromagnetically below the critical field, *H*_C_ (0.015 T), and aligning with the external field above *H*_C_. This implies that the choice of organic pillaring ligand within the Cu_3_(CO_3_)_2_(*x*)_3_·2ClO_4_ family may indeed have a profound effect on the magnetic ground state in these systems, which warrants further investigation from the perspective of the design of materials.

Thus, here we present a comprehensive investigation of the structure and properties of two previously reported MOFs belonging to this family, MOF-*bpe* and MOF-*bpy*, as well as a novel system, MOF-*azpy*, where *azpy* = 4,4′-azopyridine. We revisit the crystal structure of MOF-*bpe*, arriving at a more chemically intuitive description of the structure of this material, and provide the first structures determined from single-crystal X-ray diffraction for MOF-*bpy* and MOF-*azpy*. Through the combination of magnetic susceptibility measurements, neutron powder diffraction data collected with high flux to long *d*-spacing, and muon-spin spectroscopy, we establish that all systems undergo long-range magnetic order and aim to understand the role of the pillaring linker in tuning the magnetic properties of this family of  metal–organic kagome magnets.

## Results and Discussion

### Determining Crystal Structures from Single-Crystal X-ray Diffraction

**Figure 2:**
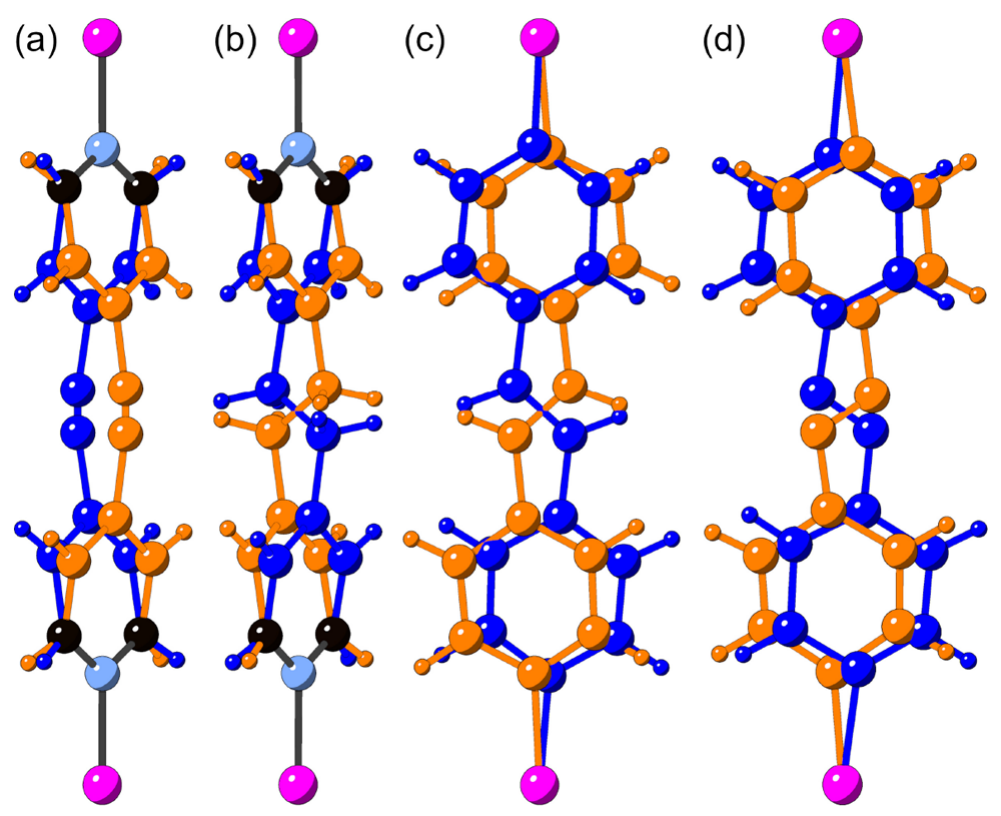
Geometry and disorder of pillaring linkers in MOF-*bpe* in (a) *P*6̅^42^ and (b) *P*3 (this work) space groups and linker disorder in (c) MOF-*bpy* and (d) MOF-*azpy* (where blue and orange colors are used to distinguish between disordered linker orientations; perchlorate anions and carbonate atoms omitted for the sake of clarity; Cu, magenta; C, black; N, light blue).

We have synthesized high-quality single crystals of MOF-*bpe* and determined its structure in the *P*6̅ space group reported in the literature.^42^ This structure is subject to extensive disorder about the 6-fold inversion axis of the weakly interacting perchlorate anions that occupy the hexagonal channels of the kagome network. There is also orientational disorder of the *bpe* linkers due to their rotational degree of freedom about the *c* axis. However, this space group assignment is unsatisfactory as it leads to an unrealistic geometry of the pillaring linkers (Figure 2a) due to the *a–b* mirror plane that bisects the linker. By determining the structure of MOF-*bpe* in this *P*6̅ model, we obtained interatomic distances between the carbon atoms of the ethylene groups in the two disordered *bpe* pillars of 0.77(2) and 0.817(19) Å, and the bond angles between the carbon atoms in the *para* position of the pyridine rings and the two ethylene carbon atoms are 168.4(8)° and 171.8(7)°.

Therefore, we explored alternative solutions to our single-crystal X-ray diffraction data for MOF-*bpe*. Of the three crystals for which diffraction data were collected, solution in the *P*3 space group consistently gave an *R*_int_ value lower than that for *P*6̅ (6.49% vs 7.31% for best fits obtained in each model). Furthermore, the effective removal of symmetry elements afforded by the *P*3 model enables assignment of the correct equivalencies in the disordered linkers (Figure 2b), leading to more realistic interatomic ethylene C–C distances of 1.43(2) and 1.44(2) Å and bond angles between the *para* carbon atoms of the pyridine rings and the ethylene carbon atoms in the range of 113–117°. The occupancies of the two disordered linker orientations in the *P*3 space group refine to 0.500(6):0.500(6). This new model implies a breathing kagome network within the *a–b* plane of MOF-*bpe* (Figure 1a), with in-plane interatomic Cu–Cu distances of 4.6249(10) and 4.7006(10) Å forming alternatingly sized triangles of Cu^2+^. This noncentrosymmetric structure exists as an inversion twin, with a Flack parameter of 0.32(5). It should be noted that the *P*3̅ space group also leads to a reasonable geometry for the *bpe* linkers in MOF-*bpe*, but with an isotropic kagome lattice. Solving in this space group, however, gave a higher *R*_int_ value (7.56%) and did not provide a stable refinement.

The crystal structure of MOF-*bpy* has been described previously^39^ through the Rietveld analysis of powder X-ray diffraction data against a modified version of the reported *P*6̅ structural model of MOF-*bpe*.^42^ Refinement of this model for MOF-*bpy* yields a nonplanar geometry for the aromatic *bpy* linker, as well as the same issues as described above for the bond angles and distances in the ethenylene group. Thus, by successfully growing single crystals of MOF-*bpy*, we have been able to determine its crystal structure in the *P*3 space group, yielding a planar geometry of the *bpy* linker as expected. This structural model of MOF-*bpy* contains the same disorder about the symmetry elements of the perchlorate anions as observed for MOF-*bpe*, as well as similar orientational disorder of the pillaring linker due to rotation about the *c* axis (Figure 2c). The occupancies of the disordered linkers refine to 0.502(5):0.498(5), and the interatomic Cu–Cu distances are 4.5798(13) and 4.6719(14) Å. The structure exists as an inversion twin, with a Flack parameter of 0.28(4).

Finally, we also find that the *P*3 model extends to a new member in this family of MOFs, MOF-*azpy*, where *azpy* = 4,4′-azopyridine. As the Flack parameter for this model [0.47(10)] indicates a racemic inversion twin, solution in the centrosymmetric *P*3̅ space group was also attempted, which gave a value for *R*_int_ (3.24%) comparable to that of the *P*3 model (3.19%). In the *P*3̅ model, however, we were unable to achieve an *R*_1_ value of <20%, indicating that *P*3 is the correct space group for MOF-*azpy*. Like MOF-*bpe* and -*bpy*, there is a high degree of disorder both in the channel-dwelling perchlorate counterions and in the pillaring linker orientation (Figure 2d), where the occupancies of the disordered linkers refine to 0.508(12):0.492(12). However, data collected for this material did not allow for anisotropic refinement of the linker atom positions, with a final *R*_1_ value of 11.12%, and interatomic Cu–Cu distances of 4.568(4) and 4.665(4) Å. A summary of the crystal structure determination for all three systems is given in Table 1.

**Table 1:** Crystallographic Data for MOF-*bpe*, -*bpy*, and -*azpy*

	MOF-*bpe*	MOF-*bpy*	MOF-*azpy*
formula	C_38_H_36_Cl_2_Cu_3_N_6_O_14_	C_38_H_30_Cl_2_Cu_3_N_6_O_14_	C_32_H_24_Cl_2_Cu_3_N_12_O_14_
*M*_r_ (g mol^–1^)	1062.25	1056.20	1062.15
crystal system	trigonal	trigonal	trigonal
space group	*P*3	*P*3	*P*3
*a* (Å)	9.3115(2)	9.2297(2)	9.2116(4)
*c* (Å)	13.3034(3)	13.3817(4)	12.9467(10)
*V* (Å^3^)	998.92(5)	987.22(5)	951.39(11)
*Z*	1	1	1
ρ_calc_ (g cm^–3^)	1.766	1.777	1.854
μ (Cu Kα) (mm^–1^)	3.766	3.811	4.006
*F*(000)	539.0	533.0	533.0
crystal size (mm^3^)	0.16 × 0.07 × 0.06	0.31 × 0.04 × 0.04	0.18 × 0.12 × 0.09
*R*_int_ (%)	6.49	3.74	3.19
goodness of fit	1.06	1.09	1.70
*R*_1_,^a^*wR*_2_^b^ [*I* > 2σ(*I*)] (%)	4.10, 10.79	4.57, 12.14	11.12, 33.23
*R*_1_,^a^*wR*_2_^b^ (all data) (%)	4.24, 11.04	4.65, 12.31	11.29, 33.79
*Δρ*_max_, *Δρ*_min_ (e Å^–3^)	0.57, −0.59	1.12, −0.75	3.03, −1.50
Flack parameter	0.32(5)	0.28(4)	0.47(10)
CCDC number	2142873	2142871	2142872

a *R*_1_ = (∑||*F*_o_| – |*F*_c_||)/∑|*F*_o_|.

b ; *w* = [σ^2^*F*_o_^2^ + (*AP*)^2^ + *BP*]^−1^, where *P* = (*F*_o_^2^ + 2*F*_c_^2^)/3.

### Extracting Magnetic Exchange Interactions from Magnetic Susceptibility

**Figure 3:**
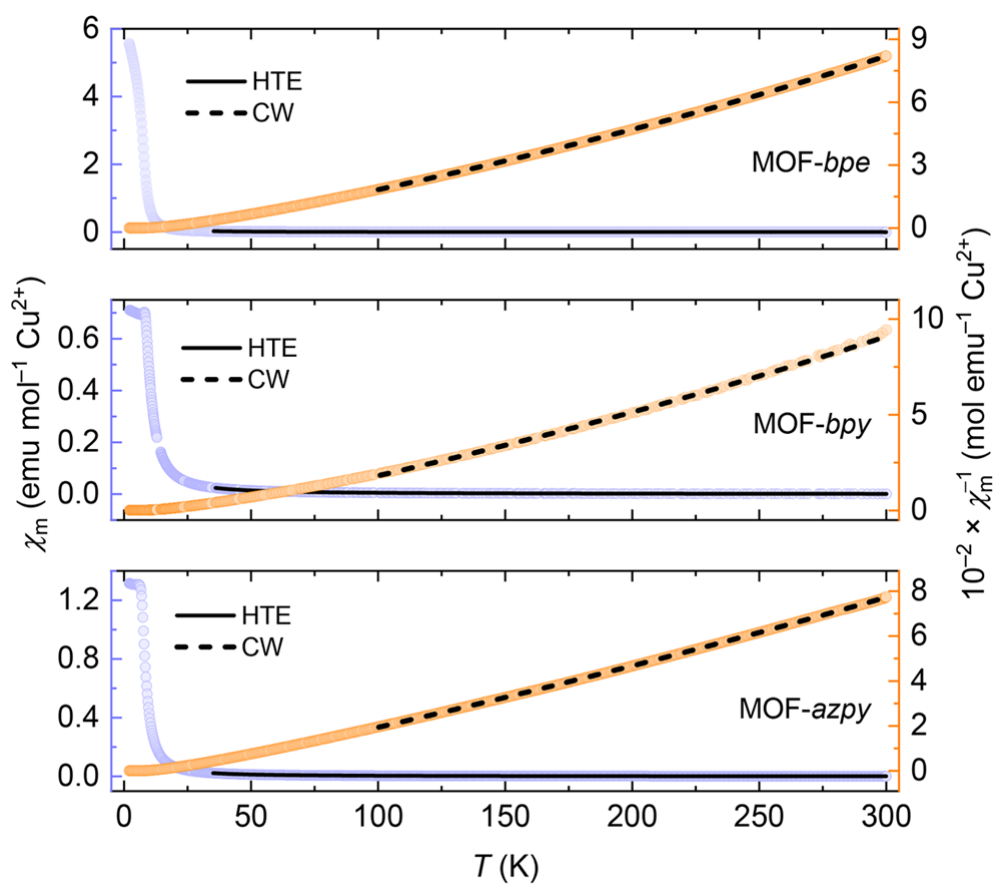
Temperature-dependent magnetic susceptibility measured in an applied field of 0.1 T for MOF-*bpe* (top), MOF-*bpy* (middle), and MOF-*azpy* (bottom). High-temperature series expansion (HTE) fitting of the molar susceptibility is shown on the left (blue) *y* axes, while Curie–Weiss (CW) fits of the inverse susceptibility are shown on the right (orange) *y* axes.

The DC molar magnetic susceptibilities (χ_m_) of MOF-*bpe*, -*bpy*, and -*azpy* are shown in Figure 3 as a function of temperature in an applied magnetic field of 0.1 T. Below 25 K, a sharp ferromagnetic upturn is observed across all three data sets. Curie–Weiss analysis of the molar magnetic susceptibilities, χ_m_ = *C*/(*T* – θ_CW_) + χ_0_, was performed for each data set to extract the Weiss temperatures (θ_CW_) and Curie constants (*C*), from which the effective magnetic moment per Cu^2+^ ion (μ_eff_) is extracted, alongside a temperature-independent susceptibility term, χ_0_, stemming from the diamagnetic background contribution. Results of this analysis are listed in Table 2.

**Table 2:** Weiss Temperatures (θ_CW_) and Effective Moments (μ_eff_) from Curie–Weiss Analysis of Inverse Susceptibility (100–300 K) and Calculated Exchange Constants (*J*_1_–*J*_3_) Obtained from High-Temperature Series Expansion of Magnetic Susceptibility (35–300 K) for MOF-*bpe*, -*bpy*, and -*azpy*

	θ_CW_ (K)	μ_eff_ (μ_B_)	χ_0_ (emu mol^–1^)	*J*_1_ (K)	*J*_2_/*J*_1_	*J*_3_/*J*_1_
MOF-*bpe*	24.99(4)	1.87(1)	–3.71(4) × 10^–4^	30.59(1)	0.93	–0.02
MOF-*bpy*	23.56(7)	1.87(9)	–5.15(9) × 10^–4^	26.15(2)	1.01	–0.02
MOF-*azpy*	22.43(4)	1.82(1)	–2.05(6) × 10^–4^	24.39(1)	1.00	–0.02

From mean field theory, one can show that the Weiss temperature extracted from the Curie–Weiss model is the sum of all exchange interactions within a material.^47^ However, the values of θ_CW_ reported in the literature for MOF-*bpe* vary considerably from −39.7 K^39^ to 60 K.^45^ This variation from net antiferromagnetic to ferromagnetic interactions may stem from sample dependence, sample hysteresis, or, possibly, a sensitivity of the Curie–Weiss fitting parameters to the temperature range over which the model is applied to the data. Thus, to examine the effect of the temperature fitting range on the extracted parameters, all Curie–Weiss fits were performed over a minimum fitting temperature, *T*_min_, varying between 15 and 200 K (Figure S1) to the data. Across all three samples, the extracted parameters were independent of the fitting range above a *T*_min_ of 100 K, with a sharp divergence in the fit parameters from a *T*_min_ of 50 K. As such, all Curie–Weiss fits were performed within the temperature range of 100–300 K. In contrast to previous studies,^39,42,46^ our extracted Weiss temperatures consistently indicate dominant ferromagnetic exchange interactions across the series. In addition, the effective moments extracted from the Curie constants are close to the  spin-only moment of 1.73 μ_B_ per Cu^2+^ (see Table 2).

**Figure 4:**
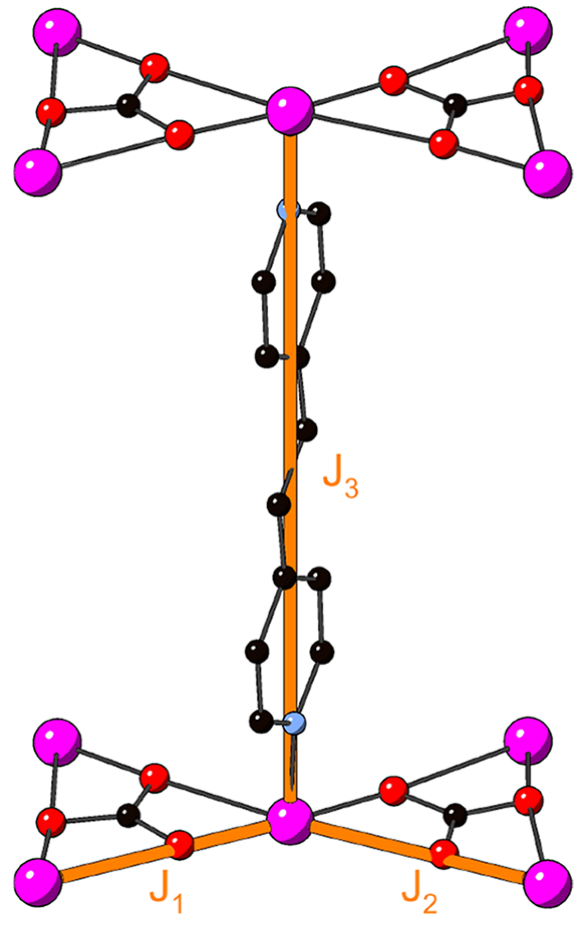
Three exchange parameters used in the high-temperature series expansion fits to magnetic susceptibility data, where *J*_1_ is the exchange over the shortest Cu–Cu distance, *J*_2_ is the exchange over the longer Cu–Cu distance in the breathing kagome model, and *J*_3_ is the interplanar Cu–Cu exchange.

Of course, a limitation in the Curie–Weiss analysis of magnetic susceptibility data is that it can yield only the net energy scale of magnetic interactions within a material. For these MOF kagome materials—in which, as described above, it is challenging to predict the nature and energy scale of the various superexchange pathways available—further insight is needed to comprehensively understand their magnetic properties. Therefore, to investigate the nature of the individual magnetic exchange interactions in MOF-*bpe*, -*bpy*, and -*azpy*, the HTE10 code^48^ was employed to compute the [6, 4] Padé approximant of the 10th-order high-temperature series expansion of an  Heisenberg breathing kagome network. Three exchange interactions were considered in this high-temperatures series expansion, with the shortest Cu–Cu distance within the kagome planes defined by magnetic exchange constant *J*_1_, the larger Cu–Cu distance in the breathing kagome model defined by *J*_2_, and the interplanar magnetic exchange interaction through the pillaring ligand described by *J*_3_ (Figure 4). As noted in the literature,^49^ high-temperature series expansion is valid only when *T* > *J*_1_, as one can see in the difference plots below *T* ≈ *J*_1_ for each system (Figure S2), and thus, the HTE fits are shown to this limit in Figure 3.

Upon application of the HTE model to magnetic susceptibility data, *J*_1_ was first extracted by fitting the data above 35 K. Subsequently, *J*_2_ and *J*_3_ were varied in 0.01 *J*_1_ steps between 0.8 *J*_1_ and 1.2 *J*_1_ and between −0.3 *J*_1_ and 0.3 *J*_1_, respectively, to inspect whether the addition of further neighbor couplings improves the overall quality of the fit. Crucially, we find that to fit the magnetic susceptibility, ferromagnetic (positive) nearest-neighbor exchange constants are required for the in-plane interactions, *J*_1_ and *J*_2_, indicating that the relevant  kagome model for these materials is a ferromagnetic one. Moreover, as shown in Table 2, the derived *J*_1_ values for MOF-*bpe*, -*bpy*, and -*azpy* are in good agreement with those predicted by DFT calculations for tridentate μ_3_-CO_3_ Cu^2+^ clusters based on their respective φ_1_ and φ_2_ angles (Figure 1c).^44^ There is a slight variation in *J*_1_ as a function of pillaring ligand; however, the driving force for this observation is not obvious from inspection of the relevant Cu–Cu distances and Cu–O···O angles across the series (Table S1). In the cases of MOF-*bpy* and -*azpy*, a *J*_2_/*J*_1_ ratio of ≈1 indicates that the anisotropy caused by the breathing kagome network in the underlying crystal structure has a negligible impact on the magnetic interactions within the kagome planes and demonstrates that the data are well described by a model of an ideal  kagome ferromagnet. However, this is not the case for MOF-*bpe*, in which the breathing nature of the kagome network appears to have a more significant effect on the overall fit, yielding a *J*_2_/*J*_1_ ratio of 0.93 and highlighting again the importance of the pillaring linker in determining the ground-state selection of this family of materials. Importantly, the high-temperature series expansion fits for all three systems are improved by the inclusion of a weak antiferromagnetic interplanar exchange interaction, *J*_3_, which—alongside possible further neighbor in-plane exchange interactions—is the likely cause of the long-range magnetic order observed in the ground states of MOF-*bpe*, -*bpy*, and -*azpy*, as detailed below.

**Figure 5:**
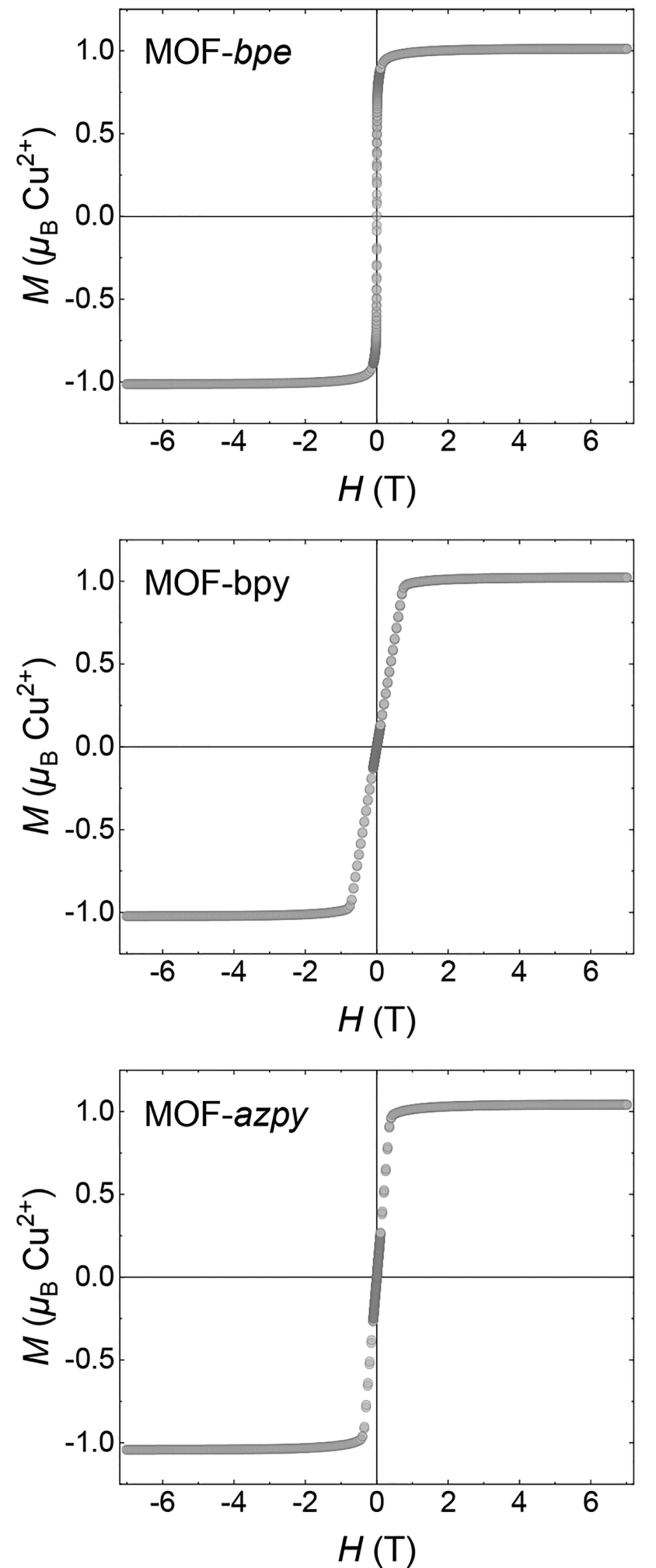
Magnetization (*M*) vs field (*H*) data collected at 2 K for samples of MOF-*bpe* (top), MOF-*bpy* (middle), and MOF-*azpy* (bottom).

To understand better the low-temperature magnetic response of each system, magnetization (*M*) versus field (*H*) data measured at 2 K for MOF-*bpe*, -*bpy*, and -*azpy* are shown in Figure 5. After calibrating for the remanent field of the magnetic property measurement system (MPMS) magnet, we did not observe magnetic hysteresis in any of the three systems, in contrast with previous findings suggesting coercive fields, *H*_C_, of 60 mT for MOF-*bpe* and 6 mT for MOF-*bpy*.^39^ Further high-resolution ultra-low-field *M* versus *H* data collected between −2.5 and 2.5 mT following nulling of the magnetic field of the MPMS place an upper limit of 0.02 mT for any coercivity (Figure S3), indicating that caution over remanent magnetic fields is required in the measurements of the low-temperature magnetic response of these materials. As one can see in Figure 5, the initial magnetization response is much larger and saturation is achieved at a lower field for MOF-*bpe* (300 mT) than for MOF-*bpy* (800 mT) and MOF-*azpy* (450 mT). In the low-field region of the magnetization of MOF-*bpy* and -*azpy*, a change in gradient is evident at a critical field (≈5 mT for MOF-*bpy* and 3 mT for MOF-*azpy*). This metamagnetic transition is not observed for MOF-*bpe* (Figure S4), indicating that the ground state of MOF-*bpe* may be distinct from those of MOF-*bpy* and -*azpy*.

### Elucidating Magnetic Structure from Neutron Powder Diffraction

Thus, to address the nature of the magnetic ground states of these materials, neutron powder diffraction (NPD) data were collected for samples of MOF-*bpe*, -*bpy*, and -*azpy* on the WISH diffractometer of the ISIS Neutron and Muon Source. Above 20 K, the diffraction data can be indexed by the *P*3 structural models determined from single-crystal X-ray diffraction and described above. For MOF-*bpe*, partial deuteration of the sample measured makes conducting a full Rietveld analysis of the data challenging (Figure S5). For MOF-*bpy*, however, an undeuterated sample was measured and Rietveld refinement of the *P*3 crystal structure to data collected at 30 K yields an excellent fit to the data, with a final *R*_wp_ of 1.85% across all five banks (Figure S6 and Table S2). While it is also fully protonated, the diffraction data for MOF-*azpy* suffer from a systematic broadening of (*hk*0) reflections, which we hypothesize is related to the orientational disorder of the pillaring linkers between the kagome layers (Figure S7).

**Figure 6:**
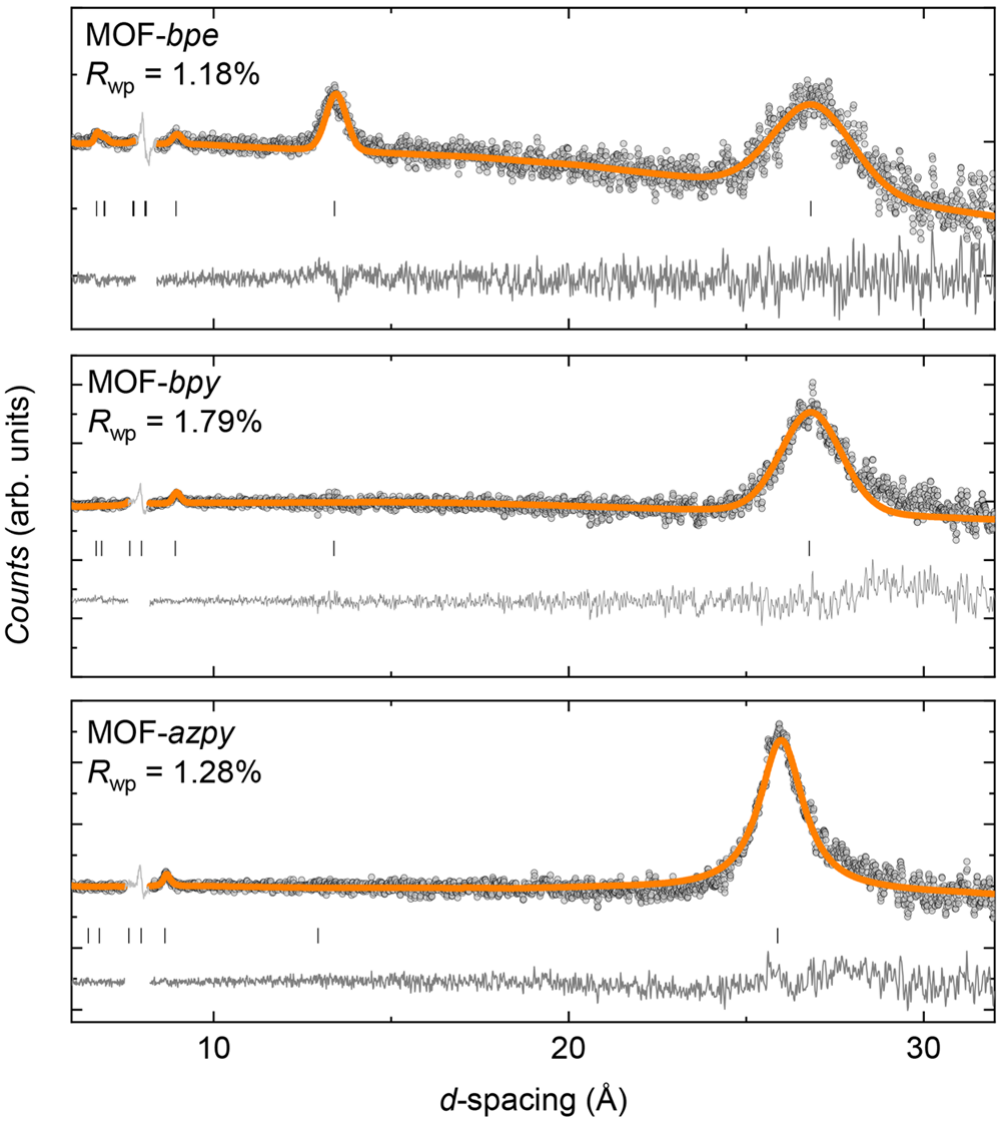
Magnetic Rietveld refinements of the constrained *P*1 model to NPD data at 1.5 K, from which data collected at 20 K (MOF-*bpe*, top), 30 K (MOF-*bpy*, middle), and 25 K (MOF-*azpy*, bottom) have been subtracted. The gray regions in the data were excluded from the fits because they are positions of strong nuclear reflections.

Nevertheless, upon cooling below 20 K, it is clear that magnetic Bragg scattering intensity develops in the NPD data sets of all three systems, which is most evident in temperature-subtracted data collected in bank 1 of the WISH diffractometer (Figure 6). The widths of the observed magnetic Bragg peaks are comparable to the instrumental resolution of bank 1 of the WISH diffractometer, suggestive of long-range magnetic order. In all three analogues, the most prominent magnetic Bragg peak is present at a *d*-spacing corresponding to twice the length of the crystallographic *c* axis, implying a magnetic propagation vector **k** = (0, 0, 0.5). Bragg intensity is also observed at a *d*-spacing corresponding to two-thirds of the *c* axis of the parent cell, corresponding to the (003) magnetic Bragg peak. In MOF-*bpe*, additional intensity is present at ≈13.3 Å, corresponding to the (002) magnetic Bragg peak. By searching for allowed magnetic structures in the Bilbao Crystallographic Server,^50−52^ we find that just one magnetic space group, *Pc*3, is consistent with the *P*3 crystal structure (or even *P*6̅, the space group originally proposed to describe MOF-*bpe* and -*bpy* as detailed above^39,42^) and **k** = (0, 0, 0.5). However, inspection of the systematic absences of Pc3 reveals that all (00*l*) magnetic peaks are forbidden within this space group. The magnetic structure described by this *Pc*3 model contains one magnetic site, a 3-fold axis, and magnetic moments that are antiparallel between adjacent planes. This means that a ferromagnetic component is not permitted by *Pc*3 symmetry, which is inconsistent with the analysis of the magnetic susceptibility data discussed in the previous section (Figure 3 and Table 2). This implies that the magnetic order in the ground states of MOF-*bpe*, -*bpy*, and -*azpy* must be described by the lower-symmetry *P*1 magnetic space group, the only subgroup of *Pc*3, thus breaking the symmetry of the underlying crystal structure.

**Figure 7:**
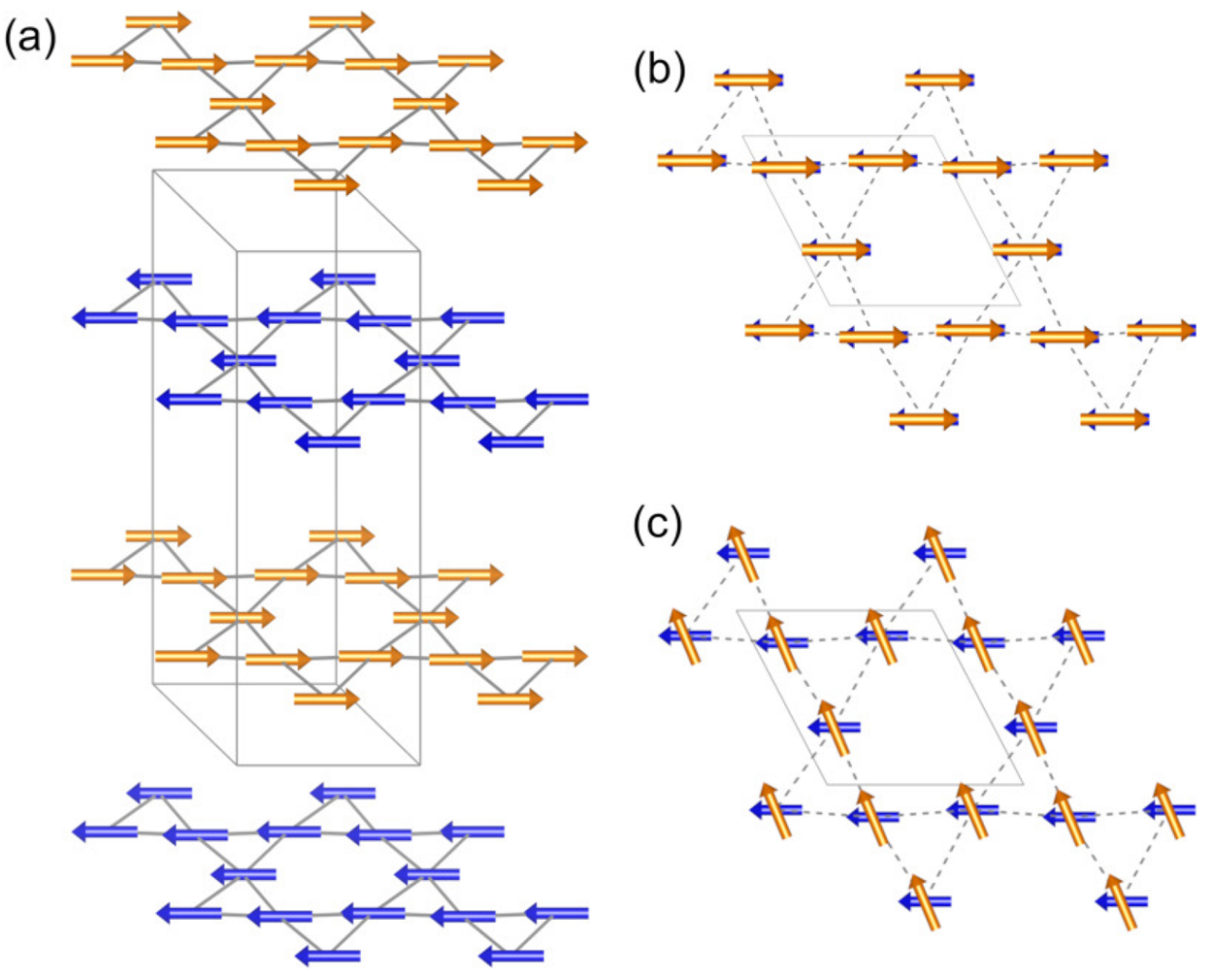
(a) In the proposed magnetic structures of MOF-*bpe*, -*bpy*, and -*azpy*, ferromagnetically aligned magnetic moments of Cu^2+^ within the kagome layers in the *a–b* plane are aligned antiferromagnetically along the *c* axis. (b) View along the *c* axis for MOF-*bpy* and -*azpy*, where the magnetic moments are perfectly antiferromagnetically aligned between adjacent layers, (c) while in MOF-*bpe*, one proposed structure is that in which the moments are canted between adjacent layers, which leads to (002) magnetic peak intensity.

This *P*1 magnetic structure has no symmetry constraints, meaning that each of the six magnetic moments within the magnetic unit cell is free to point in any direction. As a result, a sensible refinement of this model against the data collected for MOF-*bpe*, -*bpy*, and -*azpy* requires a number of constraints. First, because only (00*l*) reflections are observed in the magnetic diffraction data, the components of the magnetic moments along the *z* direction can be fixed to zero, as the (00*l*) peaks do not yield information about the moment directions out of the kagome *a–b* plane. Second, because in MOF-*bpy* and -*azpy* there is negligible magnetic intensity at the (002) position, the moments on adjacent layers must be equal and opposite such that the magnetic moments are completely antiferromagnetically aligned between neighboring kagome layers (Figure 7a,b). Within the kagome layers, unambiguously determining the precise moment directions is not possible with so few Bragg peaks. However, the simplest magnetic structure solution is one in which the intralayer exchange is fully ferromagnetic, which is consistent with our analysis of the magnetic susceptibility data presented in the previous section. Thus, for the magnetic structure refinements of MOF-*bpy* and -*azpy*, the magnetic moments were constrained to be equal and opposite within the adjacent planes, and only the moments along the *x* direction were refined. This results in total refined magnetic moments of 0.497(4) and 0.495(3) μ_B_ on each Cu^2+^ for MOF-*bpy* and -*azpy*, respectively, and yields a good fit to each data set (Figure 6). In contrast, in MOF-*bpe*, the (002) magnetic Bragg peak is present, implying that there is a difference in the moment magnitudes or spin directions between the two kagome layers within the doubled unit cell. Thus, a possible solution to the magnetic structure of MOF-*bpe* is one in which the intraplane exchange is ferromagnetic and the interplane exchange has some antiferromagnetic character, but there is misalignment between the moments on adjacent layers, which yields magnetic moments of approximately 0.5 μ_B_ per Cu^2+^ site (Figure 7c). This larger ferromagnetic component in the magnetic structure of MOF-*bpe* thus accounts for its distinct low-field magnetization data at 2 K in comparison with MOF-*bpy* and -*azpy* (Figure S4).

Our observation that MOF-*bpe*, -*bpy*, and -*azpy* undergo long-range magnetic order to a ground state of ferromagnetic kagome layers coupled antiferromagnetically contrasts with the conclusions of the most recent study of MOF-*bpe*.^46^ There it was proposed that frustration arises from a competition between nearest-neighbor ferromagnetic and next-nearest-neighbor antiferromagnetic exchange within the kagome layers, under the assumption that the large interplanar distance precluded magnetic exchange between them.^46^ In that report, the absence of magnetic Bragg peaks in NPD data was reported, implying strong frustration and no long-range magnetic order, which is in direct contrast to the data shown in Figure 6. However, the observation of the magnetic Bragg scattering from MOF-*bpe*, -*bpy*, and -*azpy* in this case is likely due to the instrumental capabilities of the WISH diffractometer,^53^ with its high flux at long *d*-spacings required to observe magnetic Bragg peaks of large unit cell systems, such as the (001) magnetic Bragg reflection that corresponds to a *d*-spacing of ≈27 Å in these materials.

The magnetic structures we elucidate here for MOF-*bpe*, -*bpy*, and -*azpy* are, however, consistent with the current understanding of other families of MOF systems containing kagome layers of Cu^2+^ cations chelated by carbonate anions.^43,54,55^ For instance, related materials with the formula Cu_3_(CO_3_)_2_(*y*)_6_·2ClO_4_ (MOF-*y*) have also been reported, in which instead of ditopic organic ligands pillaring kagome layers, the monodentate ligand 4-aminopyridine (*apy*)^54^ or 2,4′-bipyridine (*bipy*)^55^ coordinates to the out-of-plane copper coordination site, resulting in isolated kagome layers. High-temperature series expansion performed on the magnetic susceptibility data of these systems gave nearest-neighbor exchange strengths of 8.96 K^54^ and 28.1 K,^55^ respectively, further supporting the hypothesis of ferromagnetic exchange within such metal–organic kagome layers. However, in the case of MOF-*bpe*, -*bpy*, and -*azpy*, it is clear that the presence of a ditopic linker between the kagome layers plays an important role in governing both the nearest-neighbor exchanges within the kagome layers and the eventual magnetic structure within the ground state. In this sense, MOF-*bpe* is an outlier among the systems investigated here in that its *J*_1_/*J*_2_ ratio of the two nearest-neighbor exchanges in the breathing kagome layers is significant and its magnetic ground state is distinct from those of MOF-*bpy* and -*azpy*. As discussed above, the reasons for these distinctions are not obvious from structure–property arguments and the relevant Cu–Cu distances and Cu–O···O angles across the series (Table S1). Therefore, perhaps the origin of the distinct behavior of MOF-*bpe* stems from its lack of electronic conjugation along the pillaring 1,2-bis(4-pyridyl)ethane linker, although future DFT calculations are needed to give further insight into this issue.

### Confirming Long-Range Magnetic Order through Muon-Spin Spectroscopy

**Figure 8:**
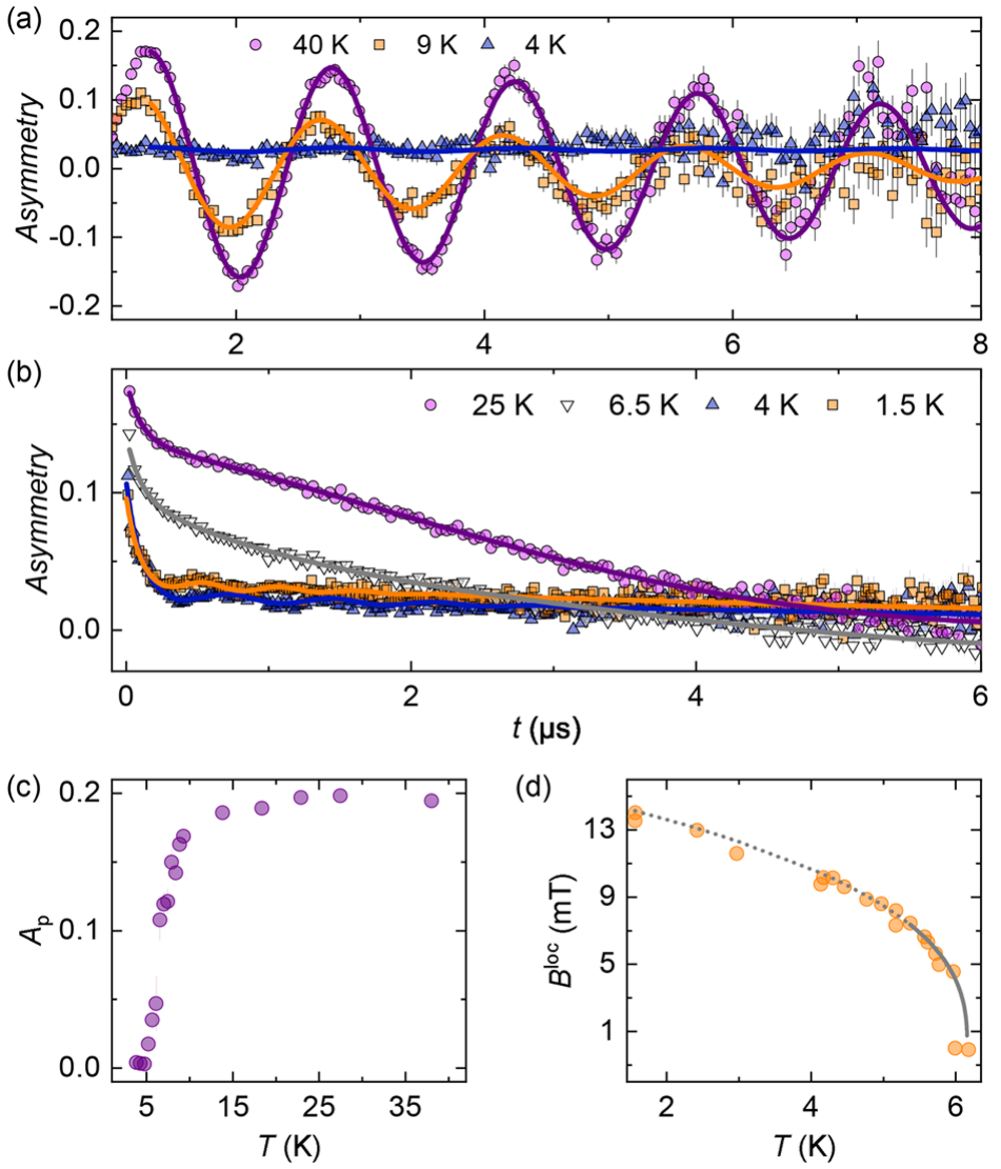
Temperature-dependent muon decay asymmetry measured for MOF-*bpe* in (a) a transverse field of 5 mT and (b) zero field, from which the (c) paramagnetic asymmetry, *A*_p_, and (d) the local magnetic field, *B*^loc^, can be determined, respectively. The solid line in panel d is a fit to the critical power law discussed in the text, and the dotted line is an extrapolation of this fit.

Finally, to firmly conclude that these MOF kagome systems undergo long-range magnetic order upon cooling below 20 K, we have performed muon-spin rotation and relaxation (μSR) measurements on a powder sample of MOF-*bpe* on the GPS instrument of the Swiss Muon Source at the Paul Scherrer Institute. Initially, room-temperature data were collected in a transverse field (TF) of 5 mT to estimate the calibration parameter between the forward and backward detector arrays (α ≈ 0.9695). Upon cooling to 40 K (Figure 8a), the TF data display an oscillation with a frequency, ν_TF_, proportional to the applied field. However, the full asymmetry expected for this instrumental configuration (≈0.282) is not observed at this temperature, indicating that there is an additional process that influences the muon-spin polarization. Indeed, in the zero-field (ZF) data, the value of the full initial asymmetry is also reduced, and there is a clear fast-relaxing component present in the data at short times that persists above 20 K (Figure 8b). One explanation for this behavior is the formation of a muonium fraction within the sample, which is a neutral state whereby the implanted muons pick up an electron and the spins of each particle are bound by a hyperfine interaction.^56,57^

To establish the magnetic transition temperature, *T*_C_, measurements were then performed in a TF of 5 mT upon cooling (Figure 8a). In this measurement geometry, the implanted muon spin is preferentially depolarized by the local internal field that develops at the muon stopping site as the sample is cooled toward its magnetic phase transition. By 4 K, one can observe in the data shown in Figure 8a that the internal local field is sufficiently strong such that all that remains is a weak background signal from muons that stop in the aluminum sample holder and thus continue to precess in the external applied TF. The TF data can be modeled at all measured temperatures using eq 1
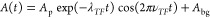
1where *A*_p_ gives the paramagnetic asymmetry of the sample, λ_TF_ accounts for the relaxation of the signal that oscillates with the applied field with frequency μ_TF_, and *A*_bg_ accounts for the background arising from the aluminum sample holder as well as the fraction of muons with their spin polarization aligned with the local magnetic field within the sample. Figure 8c shows the temperature dependence of *A*_p_, which rapidly drops below 10 K and indicates *T*_C_ ≈ 5 K, where the paramagnetic volume fraction is zero. To gain further insight into the internal field that develops in MOF-*bpe* upon cooling toward *T*_C_, a series of temperature-dependent data were collected in ZF through the magnetic transition (Figure 8b). At all temperatures, the fast-relaxing component observed at short times in the ZF data persists and is attributed to the formation of a muonium fraction as described above. At 25 K, an additional slow-relaxing component is observed, which reflects the muon-spin relaxation caused by either the nuclear magnetic moments in MOF-*bpe* or the magnetic moment fluctuations of the Cu^2+^ ions in the paramagnetic state. Thus, above *T*_C_, the ZF data can be modeled simply as the sum of two exponential relaxation terms. Upon cooling further toward *T*_C_, however, spontaneous oscillations clearly develop in the data, providing firm evidence for the onset of long-range magnetic order and the presence of an internal magnetic field about which the implanted muon spins precess. ZF data collected below *T*_C_ were thus successfully modeled by eq 2
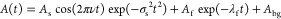
2where *A*_f_ is the fraction of the fast-relaxing component present with the relaxation rate, λ_f_, whereas *A*_s_ represents the fraction of muons that do not form muonium and thus precess in the internal field with frequency, ν, which is proportional to the local magnetic field at the muon stopping site, *B*^loc^, arising from the ordered Cu^2+^ moments, where ν = (γ_μ_/2π)*B*^loc^. The Gaussian damping term σ_s_ allows for a possible distribution of local fields or stopping sites within the structure of MOF-*bpe*, and *A*_bg_ once again gives the background signal. Figure 8d shows the temperature dependence of *B*^loc^, which follows the critical power law *B*^loc^ = *B*_0_^loc^(1 – *T*/*T*_C_)^β^. Fitting this expression to the data points collected within 10% of *T*_C_ yields a *B*_0_^loc^ of 15.7(2.5) mT, a *T*_C_ of 6.16(1) K, and a critical exponent β of 0.37(7). This critical exponent is closer to that expected for three-dimensional Heisenberg (β = 0.367),^58,59^ XY (β = 0.348),^59,60^ or Ising (β = 0.326) models^58,59^ than that expected for a two-dimensional XY (β = 0.23)^61,62^ or Ising (β = 0.125) model.^58,59,62^ Thus, the ZF μSR data presented here provide clear evidence that MOF-*bpe* undergoes long-range, three-dimensional magnetic order at a *T*_C_ of 6.16(1) K, refuting previous reports that the magnetic ground state of this family of materials lacks conventional long-range magnetic order.^46^

## Conclusions

In summary, the Cu_3_(CO_3_)_2_(*x*)_3_·2ClO_4_ (MOF-*x*, where *x* = *bpy*, *bpe*, and *azpy*) family of metal–organic frameworks containing kagome layers of  Cu^2+^ ions has been synthesized and investigated via single-crystal X-ray diffraction, magnetic susceptibility, neutron powder diffraction, and muon-spin spectroscopy. Single crystals of all three systems were successfully grown, allowing for a re-evaluation of the crystal structure of this family of materials. The structure of MOF-*bpe* was determined in the *P*3 space group, providing a more chemically intuitive geometry for the pillaring *bpe* linkers than in the reported *P*6̅ solution.^42^ The structures of MOF-*bpy* and -*azpy* were also determined in this space group, which features a breathing kagome network of alternately sized triangles of Cu^2+^ ions. Curie–Weiss and high-temperature series expansion analyses of magnetic susceptibility data indicate that the nearest-neighbor exchange interactions within the kagome planes of MOF-*bpe*, -*bpy*, and -*azpy* are all ferromagnetic, with an antiferromagnetic coupling between adjacent kagome layers through the pillaring organic ligand. This contrasts with previous studies on this family of materials, which have postulated ferromagnetic exchange between  kagome antiferromagnet layers.^39,42,46^ However, NPD data collected over a wide *d*-spacing range confirm this new model for the magnetic ground states of this family of MOFs is correct, with a doubled magnetic unit cell along the *c* axis indicating that antiferromagnetic exchange is facilitated between the kagome layers through the organic ligands. Finally, muon spectroscopy measurements support this key conclusion, demonstrating that MOF-*bpe* undergoes long-range, three-dimensional magnetic order at a *T*_C_ of 6.16(1) K, supporting our model for the magnetic ground state of this wider family of materials.

The conclusion that the magnetic ground state of the Cu_3_(CO_3_)_2_(*x*)_3_·2ClO_4_ family of MOFs realizes an  kagome ferromagnet is an important one, as it opens up new routes to search for topological states of matter predicted to arise from this model in MOF systems. Future investigations of this family of materials could, therefore, include both phonon and magnon measurement and calculation to explore the possibility of the emergence of topological excitations.^14,63^ In this regard, the breathing nature of the ferromagnetic kagome lattice is an interesting additional parameter of the underlying magnetic model realized in these systems, which may ultimately be tuned as a function of the pillaring linker between the kagome layers.^64^ Finally, it is clear from the numerous conflicting reports on the magnetic structures of these^39,42,45,46^ and related MOF materials^14,33,43^ preceding this work that the nature of magnetic superexchange in metal–organic systems is highly nontrivial. Here, the elucidation of these interactions has required the combination of multiple characterization methods, but our understanding could be deepened further through the availability of larger single crystals to allow, for example, the collection of single-crystal neutron diffraction data, as well as underpinning DFT calculations of the electronic structure and magnetic exchange interactions that govern the ground state of this family of materials.

## Methods

### Synthesis of Materials

Single crystals of Cu_3_(CO_3_)_2_(*x*)_3_·2ClO_4_ (MOF-*x*), where *x* is *bpe* [1,2-bis(4-pyridyl)ethane, C_12_H_12_N_2_], *bpy* [1,2-bis(4-pyridyl)ethylene, C_12_H_10_N_2_], or *azpy* (4,4′-azopyridine, C_10_H_8_N_4_), were prepared via a modified Solvay process.^65^ For MOF-*bpe*,^42^ Cu(ClO_4_)_2_·6H_2_O (550 mg, 1.51 mmol) and *bpe* (270 mg, 1.51 mmol) were added to aqueous NH_3_ (15%, 200 cm^3^). The mixture was stirred until fully dissolved and left to slowly evaporate over 3 days before a deep purple product was isolated by filtration and washed with H_2_O (3 × 50 cm^3^) and MeOH (3 × 50 cm^3^). MOF-*bpe* (441 mg, 0.42 mmol, 84%) was isolated as purple hexagonal plate-shaped crystals. CHN elemental analysis data were measured on a Thermo Scientific FlashSmart CHNS/O Elemental Analyzer, indicating sample purity from the expected values for Cu_3_(CO_3_)_2_(*bpe*)_3_·2ClO_4_: C, 43.0; H, 2.4; N, 7.9; found, C, 43.0; H, 2.4; N, 7.9. For single crystals of MOF-*bpy*, Cu(ClO_4_)_2_·6H_2_O (66 mg, 0.18 mmol) and *bpy* (33 mg, 0.18 mmol) were dissolved in a mixture of aqueous NH_3_ (35%, 3.75 cm^3^), methanolic NH_3_ (7 N, 4 cm^3^), and MeOH (7.25 cm^3^). The mixture was stirred until fully dissolved and left to slowly evaporate over 7 days before a deep blue product was isolated by filtration and washed with H_2_O (3 × 20 cm^3^) and MeOH (3 × 20 cm^3^). MOF-*bpy* (48 mg, 0.05 mmol, 76%) was isolated as purple hexagonal plate-shaped crystals with the expected CHN analysis: C, 43.2; H, 2.9; N, 8.0; found, C, 43.2; H, 2.9; N, 7.8. MOF-*azpy* was prepared by dissolving Cu(ClO_4_)_2_·6H_2_O (185 mg, 0.5 mmol) in aqueous NH_3_ (35%, 7 cm^3^) and H_2_O (5 cm^3^). To this was added dropwise a solution of *azpy* (92 mg, 0.5 mmol) in EtOH (5 cm^3^) and aqueous NH_3_ (35%, 7 cm^3^). The resultant black solution was stirred for 30 min, allowed to evaporate slowly for 12 days before a black product was isolated via filtration, and washed with H_2_O (3 × 20 cm^3^) and EtOH (3 × 20 cm^3^). MOF-*azpy* (152 mg, 0.14 mmol, 87%) was isolated as black needle-shaped crystals with the expected CHN analysis: C, 33.7; H, 2.5; N, 15.8; found, C, 33.3; H, 2.5; N, 15.8.

### Single-Crystal X-ray Diffraction Measurements

Single-crystal X-ray diffraction (SCXRD) data were collected for each sample on an Agilent SuperNova diffractometer with an AtlasS2 CCD detector at 100 K with Cu Kα radiation (λ = 1.54184 Å). The collection of data was driven and processed, and an absorption correction was applied using CrysAlisPro. The structures were determined by direct methods using the ShelXS package^66^ and refined by a full-matrix least-squares technique based on *F*^2^ using the ShelXL package^67^ in Olex2.^68^ All hydrogen atoms were fixed as riding models, and their isotropic thermal parameters, *U*_iso_, were based on the *U*_eq_ of the parent atom. The bond distances and angles in the disordered perchlorate anions were restrained with RIGU bond restraints, and their occupancies were fixed. For MOF-*bpe* and -*azpy*, it was not possible to refine the perchlorate anions anisotropically due to low data to parameter ratios caused by weak diffraction. In MOF-*azpy*, the linker atoms and carbonate carbon atoms also could not be refined anisotropically. Full details of the data collection and structural refinements of MOF-*bpe*, -*bpy*, and -*azpy* are listed in Table 1.

### Magnetometry Measurements

Temperature-dependent DC magnetic susceptibilities for MOF-*bpe* and -*azpy* were collected on a Quantum Design Magnetic Properties Measurement System (MPMS3) with a SQUID magnetometer. Samples (15 mg) were packed into gelatin capsules and loaded into clear plastic straw sample holders. For each sample, data were measured in applied fields of 0.05, 0.1, and 1 T over a temperature range of 2–300 K. The temperature-dependent magnetic susceptibility for MOF-*bpy* was collected on a Quantum Design Physical Property Measurement System (PPMS) with vibrating sample magnetometer (VSM). Fifteen milligrams of MOF-*bpy* was packed into a plastic capsule and loaded into a sample holder. Data were measured in an applied field of 0.1 T over a temperature range of 2–300 K. Prior to analysis, a temperature-independent diamagnetic contribution, χ_D_, was subtracted from each data set (5.16 × 10^–4^, 4.98 × 10^–4^, and 4.41 × 10^–4^ emu mol^–1^ for MOF-*bpe*, -*bpy*, and -*azpy*, respectively), which were calculated using tabulated values of Pascal’s constants.^69^ For magnetization versus field measurements, 15 mg samples of MOF-*bpe*, -*bpy*, and -*azpy* were packed into gelatin capsules and loaded into clear plastic straw sample holders, as described above. Data were collected in DC mode on an MPMS3 with a SQUID magnetometer at 2 K between −7 and 7 T. A calibration was performed using a Pd standard to correct for the remanent field in the magnet. Ultra-low-field measurements were obtained using a Quantum Design’s ultra-low-field option on the MPMS3, which can actively cancel the residual magnetic field in the superconducting solenoid. These measurements were obtained in vibrating sample magnetometer (VSM) mode between −2.5 and 2.5 mT. Shape corrections of 1.072 in DC mode and 1.145 in VSM mode were applied to magnetization data corresponding to a cylinder with a diameter of 5 mm and a height of 2 mm, as our best estimate to account for the shape of the sample within the gelatin capsule.

### Neutron Powder Diffraction Measurements

Neutron powder diffraction (NPD) data were collected on the WISH diffractometer of the ISIS Neutron and Muon Source at the Rutherford Appleton Laboratory. Samples (1 g) were packed into cylindrical vanadium cans, and data were collected at 1.5 K for each sample, as well as at 20 K (MOF-*bpe*), 30 K (MOF-*bpy*), and 25 K (MOF-*azpy*). The measured sample of MOF-*bpe* was ≈85% deuterated (ISIS Deuteration Facility) leading to a lower background than for MOF-*bpy* and -*azpy*, but also creating challenges with the subsequent structural refinement. Thus, MOF-*bpy* and -*azpy* were measured undeuterated. Rietveld analysis for data collected at higher temperatures was conducted using the GSAS-II package.^70^ All magnetic Rietveld refinements were completed using FullProf.^71^ In the structural refinement of MOF-*bpy*, the two disordered *bpy* linker orientations were defined as rigid bodies within the GSAS-II software, and their respective origins and rotation angles were allowed to refine freely within the unit cell. The thermal motion was refined isotropically and constrained across the atoms in each rigid body. Atomic positions and *U*_iso_ values for the perchlorate moieties were also fixed due to the significant disorder that they display. To further simplify the refinement, the isotropic atomic displacement parameters, *U*_iso_, were constrained across the carbonate ligands. This *P*3 structure describes the data well, with an *R*_wp_ of 1.85%, and there is no evidence for any symmetry lowering at this temperature or at 1.5 K.

### Muon-Spin Spectroscopy Measurements

Muon-spin relaxation and rotation (μSR) spectra for MOF-*bpe* were recorded on the General Purpose Surface-Muon (GPS) instrument of the Swiss Muon Source at Paul Scherrer Institute. A 1 g sample was contained in an aluminum foil packet and loaded into a ^4^He cryostat. Measurements were taken in zero field (ZF) and transverse field (TF) using the up and down detectors of the GPS instrument, with the initial muon spin rotated upward by 50°. This configuration leads to larger asymmetry and faster counting times compared to those of the forward and backward detectors. All data were analyzed using the musrfit program.^72^

## Supporting Information Available

Further details about the analysis of magnetic susceptibility and NPD data (PDF)

## Accession Codes

CCDC 2142871–2142873 contains the supplementary crystallographic data for this paper. These data can be obtained free of charge via www.ccdc.cam.ac.uk/data_request/cif, or by emailing data_request@ccdc.cam.ac.uk, or by contacting The Cambridge Crystallographic Data Centre, 12 Union Road, Cambridge CB2 1EZ, UK; fax: +44 1223 336033.
