# Supplementary material for: Uncovering the [image] Kagome Ferromagnet within a Family of Metal–Organic Frameworks
Source: Chem Mater. 2022 Jun 9;34(12):5409–21. doi: 10.1021/acs.chemmater.2c00289 (PMC9490827; doi:10.1021/acs.chemmater.2c00289)
Supplement: Supplementary file 1 — cm2c00289_si_001.pdf [file cm2c00289_si_001.pdf]

Uncovering the $S = \frac{1}{2}$ Kagome Ferromagnet Within a Family of Metal-Organic Frameworks

Supporting Information

Samuel A. Ivko,[a] *Katherine Tustain,*[b] *Tristan Dolling,*[a] *Aly Abdeldaim,*[a,c] *Otto H. J. Mustonen,*[a] *Pascal Manuel,*[c] *Chennan Wang,*[d] *Hubertus Luetkens,*[d] *and Lucy Clark*[a]

[a] School of Chemistry, University of Birmingham, Birmingham B15 2TT UK

[b] Department of Chemistry and Materials Innovation Factory, University of Liverpool, Liverpool, L7 3NY UK

[c] ISIS Neutron and Muon Source, Rutherford Appleton Laboratory, Didcot, OX11 0QX UK

[d] Swiss Muon Source, Paul Scherrer Institut, Villigen, 5232, Switzerland

Email: l.m.clark@bham.ac.uk

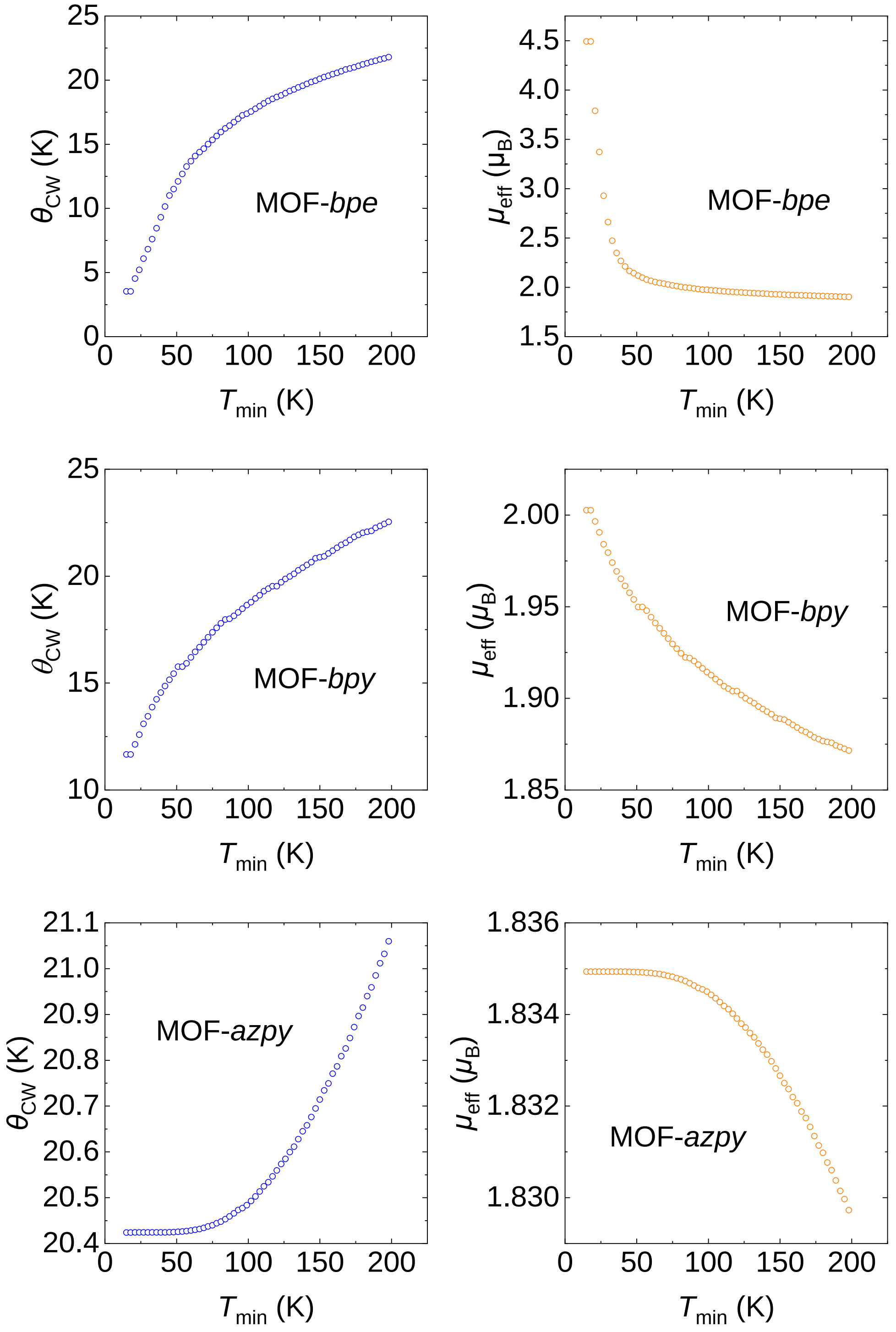

Figure S1 Derived θ_{CW} and μ_{eff} values as a function of minimum fitting temperature, T_{min}, in Curie-Weiss analysis of inverse magnetic susceptibility for MOFs -*bpe*, -*bpy* and -*azpy*.

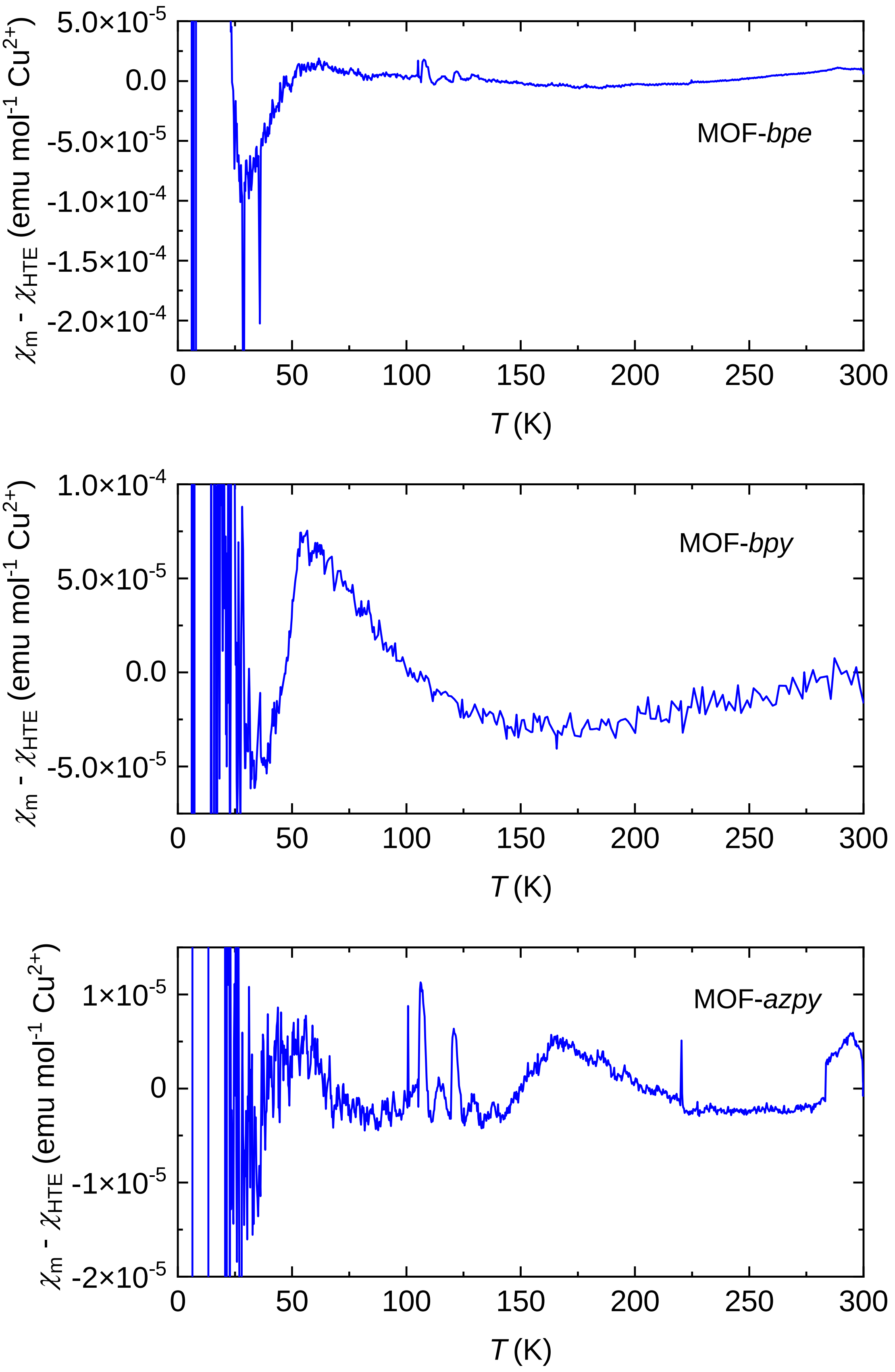

Figure S2 Difference plots between measured molar susceptibility (χ_m) and high temperature expansion fits (χ_{HTE}) for MOF-*bpe*, MOF-*bpy* and MOF-*azpy* demonstrating good fitting at $T > J_1$.

Table S1 Nearest-neighbour exchange values J_1 and J_2 derived from high-temperature series expansion analysis, along with Cu-Cu distances and Cu-O···O angles (φ_1 and φ_2) for MOFs *-bpe*, *-bpy* and *-azpy*.

Compound	MOF-*bpe*	MOF-*bpy*	MOF-*azpy*
J_1 (K)	30.59(1)	25.39(2)	24.39(1)
Cu-Cu distance (Å)	4.6249(10)	4.5798(13)	4.568(4)
φ_1 (°)	78.2(3)	76.9(3)	76.9(8)
φ_2 (°)	221.8(3)	223.1(3)	223.1(3)
J_2/J_1	0.93	0.90	1.00
Cu-Cu distance (Å)	4.7006(10)	4.6719(14)	4.665(4)
φ_1 (°)	82.2(3)	81.4(3)	78.7(9)
φ_2 (°)	217.8(3)	218.6(3)	221.3(9)

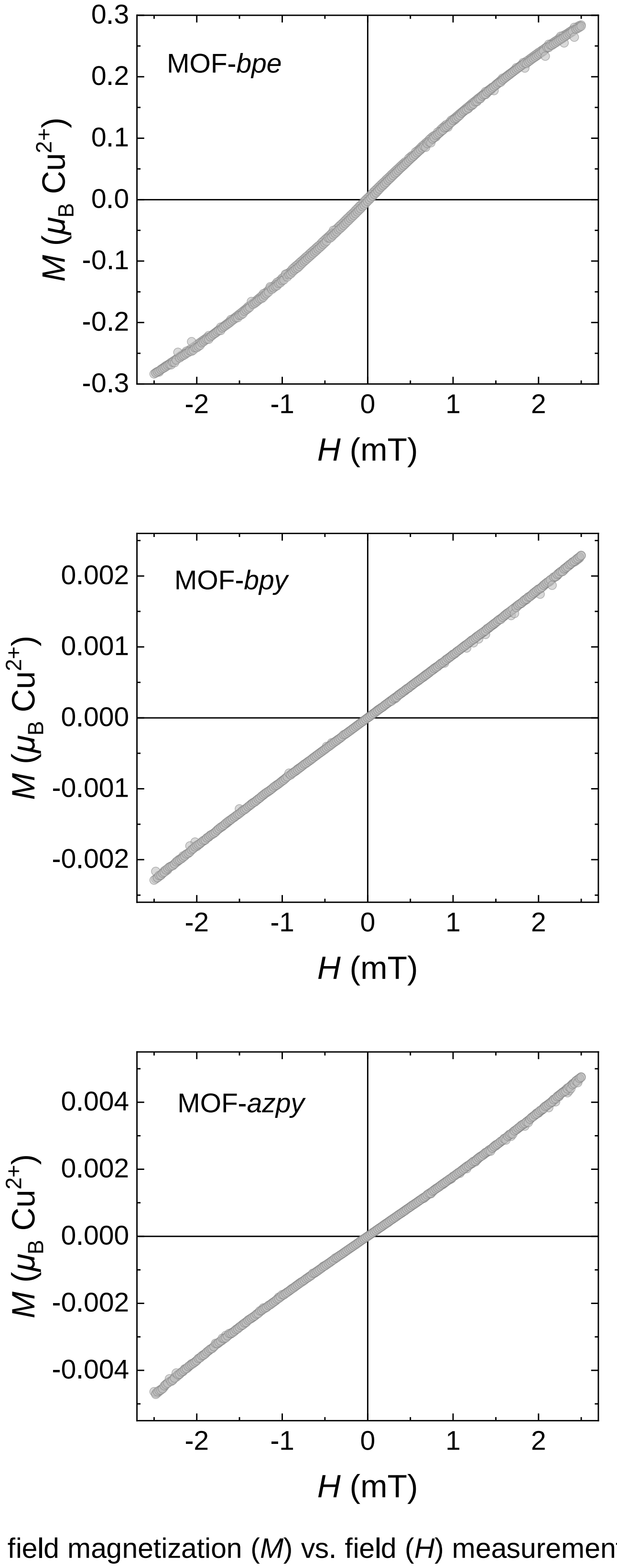

Figure S3 Ultra-low field magnetization (*M*) vs. field (*H*) measurements at 2 K for MOF-*bpe*, MOF-*bpy* and MOF-*azpy*.

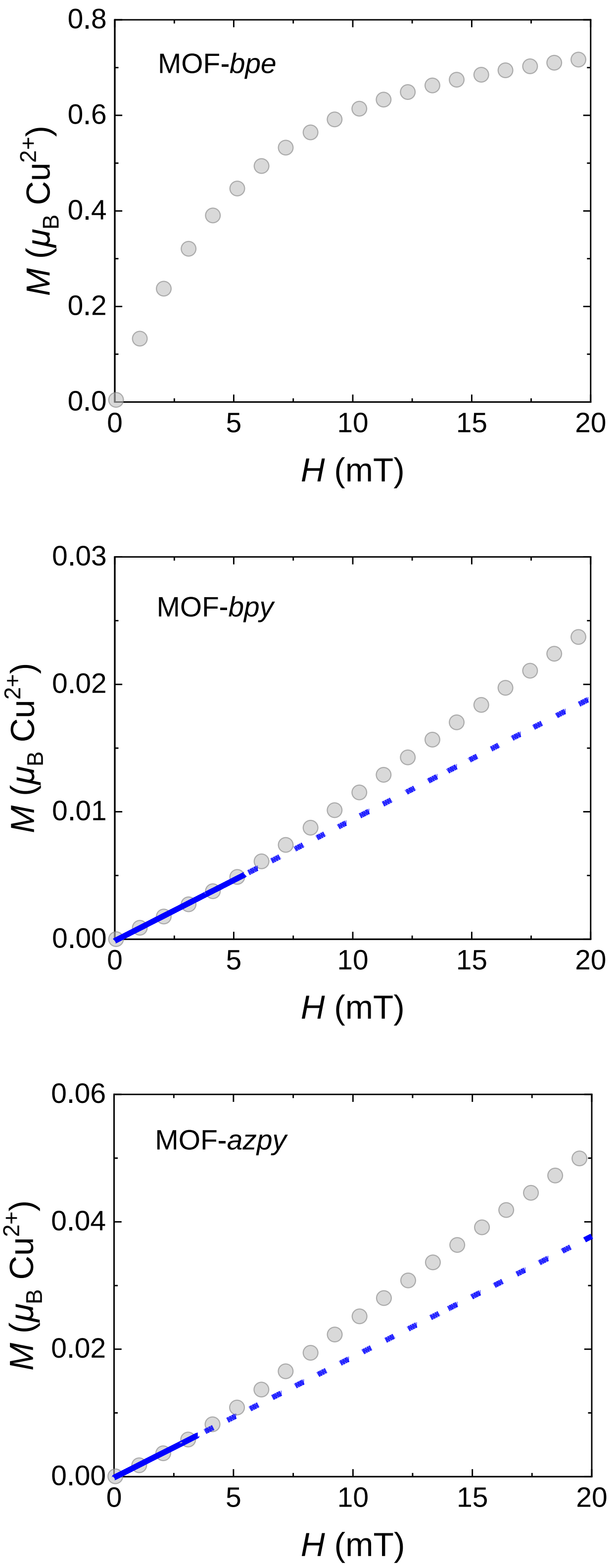

Figure S4 Low field region of initial magnetization at 2 K for MOF-*bpe*, MOF-*bpy* and MOF-*azpy* showing metamagnetic transition above critical field for MOF-*bpy* and MOF-*azpy* (dashed lines are extrapolations of fits), and lack of observable transition and rapid magnetization for MOF-*bpe*.

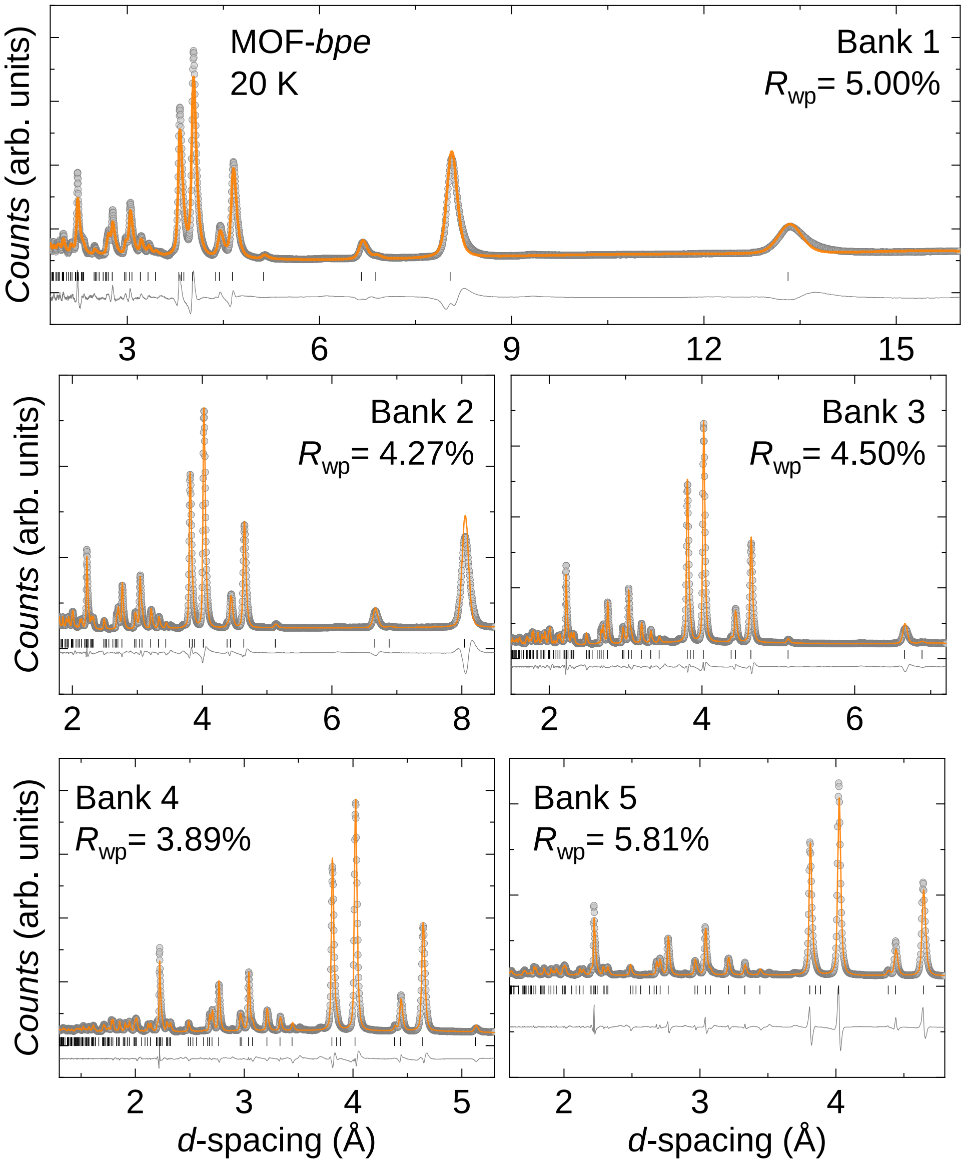

Figure S5 Le Bail refinement of the *P*3 structure of MOF-*bpe* to NPD data collected on the WISH diffractometer at 20 K (R_{wp} = 4.69%). The sample was partially deuterated, meaning that a reliable Rietveld refinement was not possible.

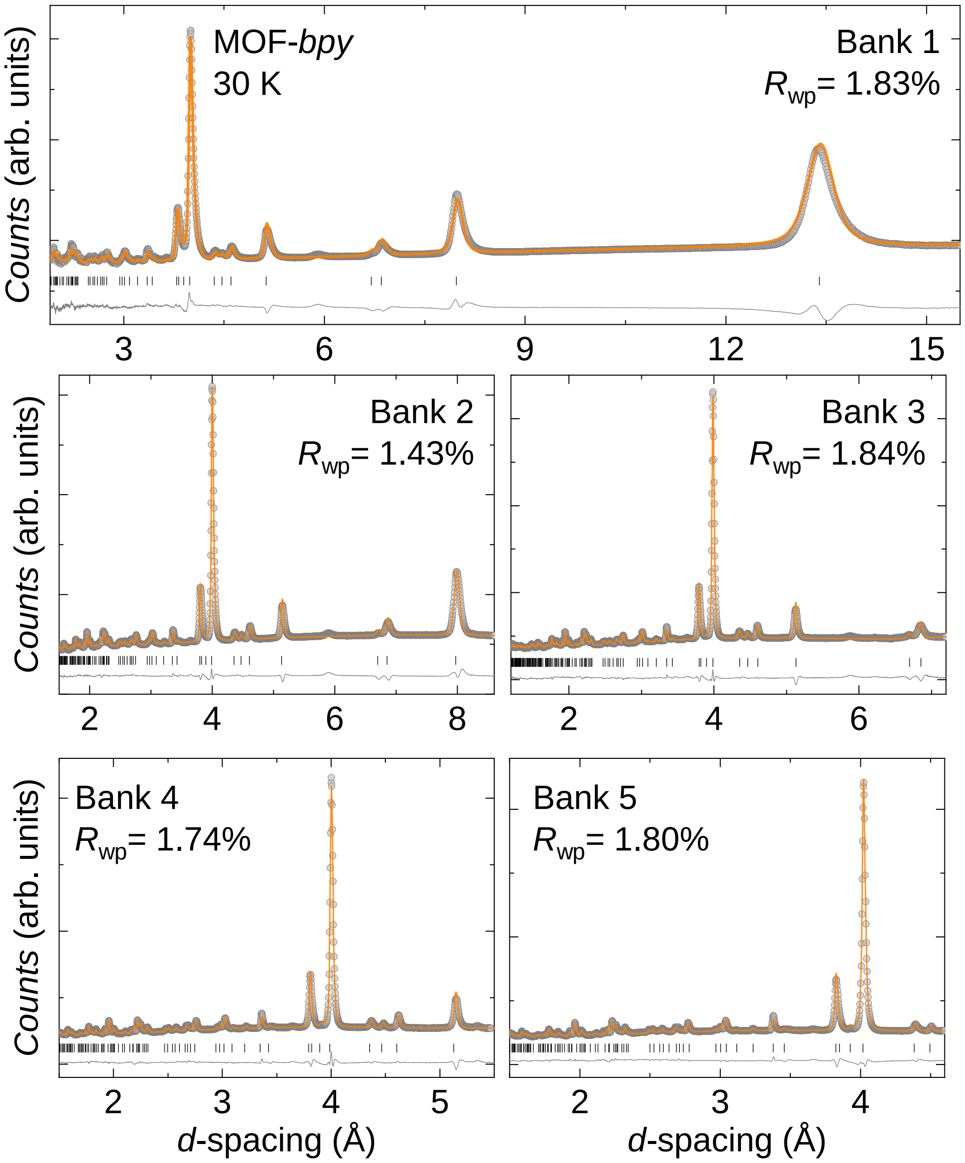

Figure S6 Rietveld refinement of the *P*3 structure of MOF-*bpy* to NPD data collected on the WISH diffractometer at 30 K. The final statistical factor across all five banks, R_{wp} = 1.85%.

Table S2 Rietveld refinement of crystal structure parameters for the *P*3 model of MOF-*bpy* fitted to NPD data collected on WISH at 30 K. Atoms denoted A and B were defined as two separate rigid bodies with refined origins of [0.155(1), 0.804(1), 0.957(1)] and [0.149(1), 0.810(1), 0.950(1)] for A and B linkers, respectively. The refined lattice parameters are $a = b = 9.2069(5)$ Å and $c = 13.3964(6)$ Å. The final statistical factors are $R_{wp} = 1.85\%$ and $\chi^2 = 1.83$.

Atom	Site	*x*	*y*	*z*	Occupancy	U_{iso}
Cu1	3*d*	0.136(2)	0.781(1)	0.454(2)	1	0.041(3)
			Carbonates			
C13	1*a*	0	1	0.451(3)	1	0.033(2)
C14	1*b*	0.33333	0.66667	0.452(3)	1	0.033
O9	3*d*	0.386(2)	0.828(2)	0.452(3)	1	0.033
O10	3*d*	0.994(2)	0.848(2)	0.451(3)	1	0.033
			Linker A			
N1A	3*d*	0.11391	0.76832	0.30777	0.5	0.020(4)
C1A	3*d*	0.97421	0.67254	0.25648	0.5	0.020
H1A	3*d*	0.87467	0.60478	0.29303	0.5	0.020
C2A	3*d*	0.25664	0.86387	0.25456	0.5	0.020
H2A	3*d*	0.35781	0.93151	0.28980	0.5	0.020
C3A	3*d*	0.65980	0.66707	0.15427	0.5	0.020
H3A	3*d*	0.86278	0.59435	0.12222	0.5	0.020
C4A	3*d*	0.25911	0.86733	0.15055	0.5	0.020
H4A	3*d*	0.36056	0.93512	0.11531	0.5	0.020
C5A	3*d*	0.10509	0.76667	0.09768	0.5	0.020
C6A	3*d*	0.09185	0.75845	0.98857	0.5	0.020
H6A	3*d*	0.84830	0.68502	0.96090	0.5	0.020
C7A	3*d*	0.21745	0.84576	0.92391	0.5	0.020
H7A	3*d*	0.32587	0.91743	0.95078	0.5	0.020
C8A	3*d*	0.19935	0.83773	0.81240	0.5	0.020
C9A	3*d*	0.05175	0.73621	0.76266	0.5	0.020
H9A	3*d*	0.95218	0.66452	0.79823	0.5	0.020
C10A	3*d*	0.34692	0.94524	0.75779	0.5	0.020
H10A	3*d*	0.44823	0.01776	0.79122	0.5	0.020
C11A	3*d*	0.33737	0.94050	0.65795	0.5	0.020
H11A	3*d*	0.43617	0.01063	0.62087	0.5	0.020
C12A	3*d*	0.05391	0.74151	0.65935	0.5	0.020
H12A	3*d*	0.95283	0.67338	0.62391	0.5	0.020
N2A	3*d*	0.19499	0.84059	0.60731	0.5	0.020
			Linker B			
N1B	3*d*	0.16690	0.81972	0.30015	0.5	0.018(3)
C1B	3*d*	0.02772	0.74792	0.24534	0.5	0.018
H1B	3*d*	0.92184	0.69321	0.27803	0.5	0.018
C2B	3*d*	0.31533	0.89799	0.25211	0.5	0.018
H2B	3*d*	0.41342	0.94987	0.29203	0.5	0.018
C3B	3*d*	0.03374	0.75054	0.14154	0.5	0.018
H3B	3*d*	0.93256	0.69910	0.10385	0.5	0.018
C4B	3*d*	0.33565	0.91057	0.15202	0.5	0.018
H4B	3*d*	0.44338	0.96926	0.12214	0.5	0.018
C5B	3*d*	0.18596	0.82842	0.82842	0.5	0.018
C6B	3*d*	0.20651	0.83842	0.98344	0.5	0.018
H6B	3*d*	0.31808	0.89350	0.95902	0.5	0.018
C7B	3*d*	0.08560	0.77845	0.91535	0.5	0.018
H7B	3*d*	0.97388	0.72285	0.93965	0.5	0.018

Continued on next page

Atom	Site	*x*	*y*	*z*	Occupancy	U_{iso}
C8B	3*d*	0.10662	0.78951	0.80703	0.5	0.018
C9B	3*d*	0.96626	0.71486	0.74728	0.5	0.018
H9B	3*d*	0.85804	0.65659	0.77638	0.5	0.018
C10B	3*d*	0.26639	0.86655	0.75963	0.5	0.018
H10B	3*d*	0.36619	0.91299	0.79817	0.5	0.018
C11B	3*d*	0.27379	0.87332	0.65409	0.5	0.018
H11B	3*d*	0.37894	0.92686	0.62149	0.5	0.018
C12B	3*d*	0.98470	0.72770	0.64580	0.5	0.018
H12B	3*d*	0.88628	0.67820	0.60597	0.5	0.018
N2B	3*d*	0.13197	0.80392	0.59923	0.5	0.018
			Perchlorates			
Cl1	3*d*	0.66400	0.33100	0.97070	0.333	0.005
O1	3*d*	0.55300	0.18600	0.91500	0.333	0.005
O2	3*d*	0.59200	0.29200	0.07400	0.333	0.005
O3	3*d*	0.65330	0.47720	0.95440	0.333	0.005
O4	3*d*	0.82120	0.35350	0.00350	0.333	0.005
Cl2	3*d*	0.66300	0.33300	0.47070	0.333	0.005
O5	3*d*	0.84300	0.45700	0.46800	0.333	0.005
O6	3*d*	0.61400	0.22400	0.38200	0.333	0.005
O7	3*d*	0.56600	0.41900	0.47600	0.333	0.005
O8	3*d*	0.63400	0.24400	0.56740	0.333	0.005

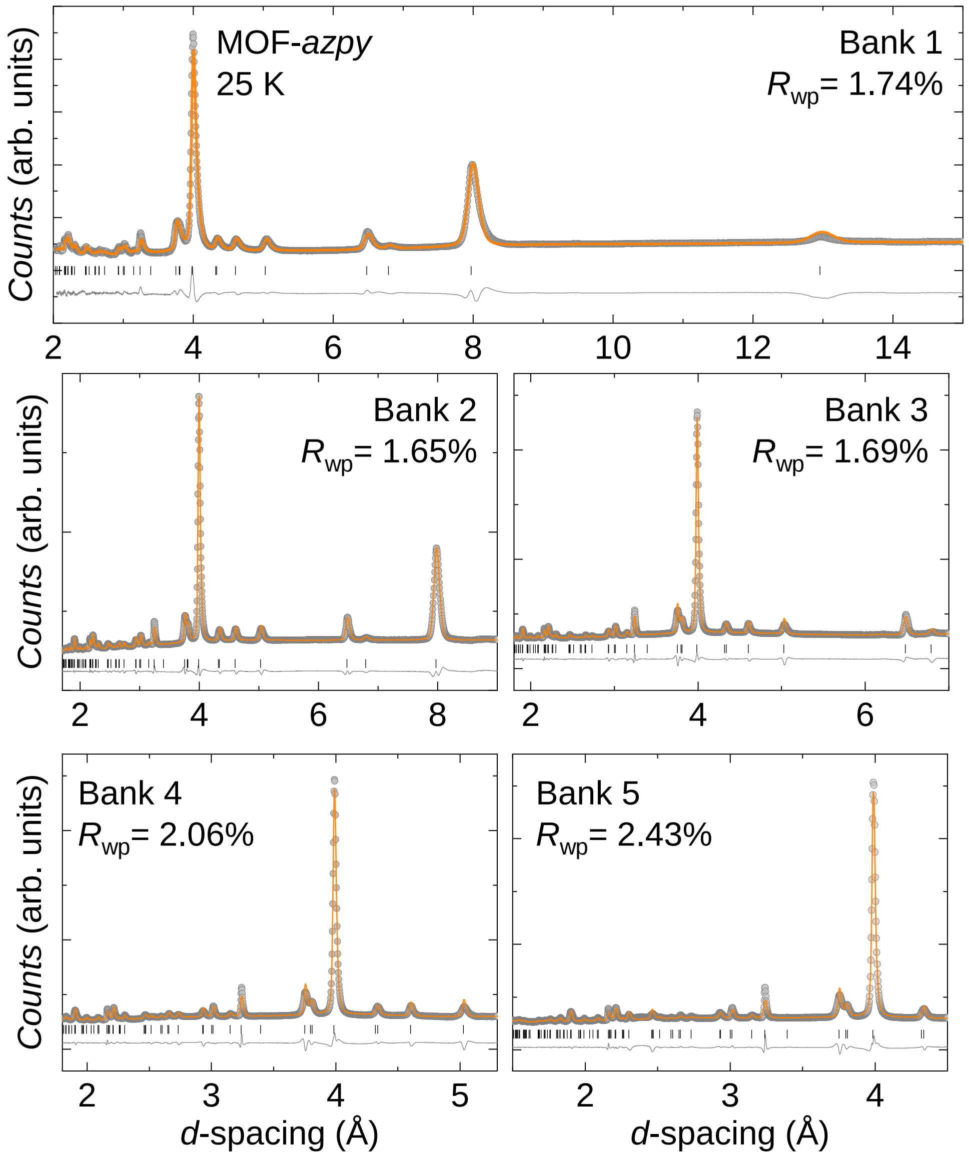

Figure S7 Le Bail refinement of the *P*3 structure of MOF-*azpy* to NPD data collected on the WISH diffractometer at 25 K (R_{wp} = 1.90%). The (00*l*) peaks are systematically sharp, meaning that a reliable Rietveld refinement was not possible.
